# Impact of co-amoxiclav versus amoxicillin on the gastrointestinal microbiota in sub-Saharan children hospitalized with pneumonia

**DOI:** 10.1038/s44259-026-00216-5

**Published:** 2026-05-22

**Authors:** Juan Pablo Rodriguez-Ruiz, Servaas Hiel, Victor Musiime, Veronica Mulenga, Hilda A. Mujuru, Shabir A. Madhi, David P. Moore, Moherndran Archary, Michelle Clements, A. Sarah Walker, Julia A. Bielicki, Michael Sharland, Surbhi Malhotra-Kumar

**Affiliations:** 1 Laboratory of Medical Microbiology, Vaccine & Infectious Disease Institute, University of Antwerp, Antwerp, Belgium; 2 Department of Paediatrics and Child Health, School of Medicine, College of Health Sciences, Makerere University, Kampala, Uganda; 3 Joint Clinical Research Centre, Kampala, Uganda; 4 University Teaching Hospital, Lusaka, Zambia; 5 Department of Paediatrics and Child Health, University of Zimbabwe College of Health Sciences, Harare, Zimbabwe; 6 South African Medical Research Council Vaccines and Infectious Diseases Analytics Research Unit, University of the Witwatersrand, Johannesburg, South Africa; 7 Wits Infectious Diseases and Oncology Research Institute, Faculty of Health Sciences, University of the Witwatersrand, Johannesburg, South Africa; 8 School of Clinical Medicine, Faculty of Health Sciences, University of the Witwatersrand, Johannesburg, South Africa; 9 Africa Health Research Institute, Durban, South Africa; 10 MRC Clinical Trials Unit at University College London, University College London, London, UK; 11 Department of Paediatric Infectious Diseases, University of Basel, Basel, Switzerland; 12 Centre for Neonatal and Paediatric Infection, City St George’s University of London, London, UK

**Keywords:** Diseases, Health care, Medical research, Microbiology

## Abstract

Community-acquired pneumonia (CAP) causes high pediatric mortality especially in sub-Saharan Africa. In a secondary analysis of samples from the PediCAP trial (ISRCTN63115131), we tracked gastrointestinal resistome and microbiota dynamics of 149 children (<6 years) from South Africa, Zambia, Zimbabwe and Uganda presenting with severe CAP. Patients received initial WHO-standard IV therapy before being randomized to continue IV therapy or step-down to oral amoxicillin or co-amoxiclav for different total treatment durations. Samples were obtained after starting IV antibiotics, at discharge, and at four weeks follow-up. Microbiota dynamics were strongly associated with age and country of origin with specific bacterial genera unique to each country. Oral step-down to either antibiotic regimen or duration did not show any differential effects on the gastrointestinal microbiota and resistome dynamics compared to continuous IV therapy. The data of this secondary analysis complement the primary PediCAP trial analysis, supporting earlier hospital discharge for paediatric CAP patients.

## Introduction

Community-acquired pneumonia (CAP) is a leading infectious cause of morbidity and mortality, especially in children under 5 years of age in sub-Saharan Africa^1^. Antimicrobial resistance (AMR) represents an increasing concern, in particular regarding lower respiratory tract infections, with 5.05 deaths per 100,000 individuals directly attributable to AMR in 2021, and three-fold higher rates in sub-Saharan Africa^2^.

Gastrointestinal microbiota research in African countries is limited, with low representation of diseases responsible for the highest morbidity and mortality^3,4^. Additionally, most paediatric microbiota studies conducted in Africa focus on limited geographic areas and often involve observational cross-sectional designs^3,4^.

The PediCAP trial (ISRCTN63115131) aimed to evaluate the impact of oral step-down to dispersible amoxicillin or co-amoxiclav tablets, and of duration of antibiotic therapy, on effectiveness in symptom resolution, safety and selection of antibiotic resistance in severe childhood CAP in Africa^5^. The trial showed that oral step-down, and 4-5 days total (intravenous (IV) plus oral) antibiotic therapy, achieved non-inferior cure rates and earlier discharge compared with 5 days IV therapy, representing a safe and effective alternative to continued IV therapy^5^.

Here, we performed a secondary sample analysis to track the dynamics of the gastrointestinal microbiota and resistome in children admitted to hospital with severe CAP in four sub-Saharan countries (South Africa, Zambia, Zimbabwe and Uganda) within the PediCAP trial (Statistical Analysis Plan in Supplementary Information). The primary objective was to estimate the effect of the different randomized treatment strategies tested in the trial (step-down with narrower oral amoxicillin vs broader oral co-amoxiclav vs continuous IV treatment, and total duration of antibiotic treatment) on AMR development and associated changes in the microbial community, given that benefits from narrower and/or shorter antibiotic courses on the resistome/microbiome are often postulated. As a secondary exploratory objective, we also identify the clinical factors influencing these dynamics.

## Results

### Study population and sequencing QC

**Fig. 1:**
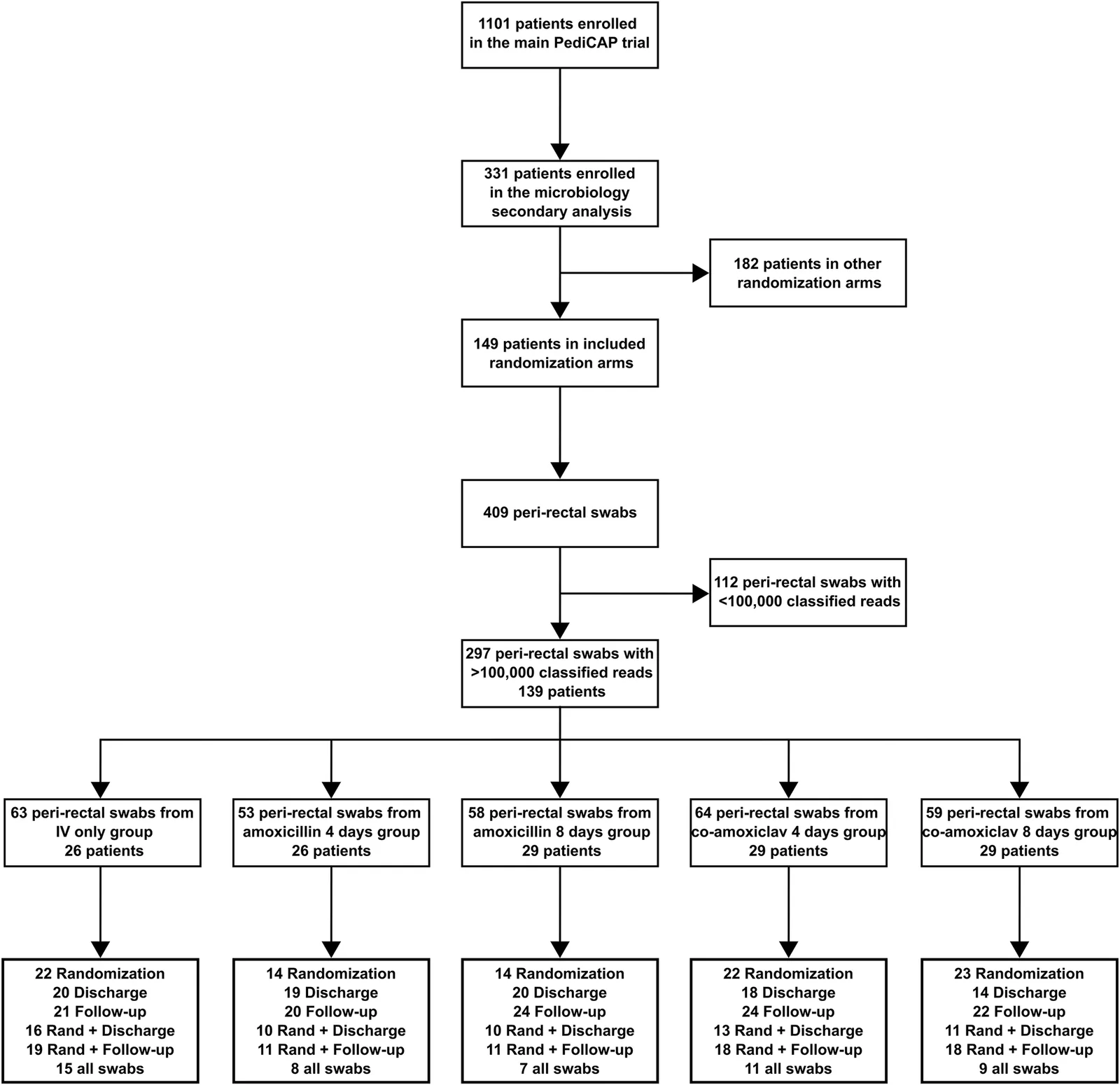
Flow chart depicting the sub-study population.

The PediCAP trial (ISRCTN63115131) enrolled 1101 children ≤6 years old admitted to hospital due to severe CAP and judged to require at least 24 h of IV antibiotic treatment^5^. Children initially received IV therapy following World Health Organization (WHO) CAP treatment guidelines; and were randomized within 24 h of initiating IV to continue IV antibiotics for five days, or step down to oral amoxicillin or oral co-amoxiclav dispersible tablets when well enough for oral medication for total treatment durations (IV+oral) between four and eight days. From all enrolled children, 331 consented to join the microbiology sub-study, involving sampling at randomization, discharge and a follow-up visit four weeks after randomization (Statistical Analysis Plan in Supplementary Information). Of these, 149 children were randomized to the treatment groups compared in this analysis: IV only, amoxicillin step-down total 4 days, amoxicillin step-down 8 days, co-amoxiclav step-down 4 days, and co-amoxiclav step-down 8 days groups (Fig. 1). The remaining 182 children were not included due to belonging to other randomization arms.

Overall, 409 (91.5%) of the expected 447 peri-rectal swabs from the included randomized groups were collected and processed, yielding a mean 9,314,689 reads, of which 22.6% were classified as human contamination and removed by the Hostile pipeline^6^, resulting in a mean 6,326,601 microbial reads per sample. Of these, a mean 5,539,302 reads were taxonomically classified using the UHGG (Unified Human Gastrointestinal Genome) database^7^, showing sequencing depth was adequate; however, samples with <100,000 classified reads were very far from plateauing in microbial species rarefaction curves (Fig. S1). Excluding the 112 (27.3%) samples with <100,000 classified reads from further analysis left sequencing data from 297 peri-rectal swabs from 139 children, relatively evenly distributed across randomized groups and timepoints (Fig. 1). Comparing with negative controls included in every DNA extraction batch, 365/4628 (7.9%; 95% confidence interval, 7.1 to 8.7%) detected species were determined to be contaminants by the decontam pipeline, and were removed from further analysis^8^.

Differences between randomized groups included, as expected, total duration of IV and oral antibiotic treatment, as well as days since the last antibiotic intake at follow-up (Table 1). When considering country of origin (Table S1), CRP values were higher in Zambia and Zimbabwe, fewer vaginal deliveries were observed in South Africa, Ugandan children received less oxygen supplementation at admission. Additionally, antibiotic intake before admission and use of non-trial antibiotics showed higher rates in Zambia, initial IV treatment was markedly different between countries, and total duration of IV treatment and hospitalization was higher in Zambia, with Zimbabwean children also being significantly younger (Table S1).

**Table 1:** Clinical characteristics associated with sub-study patients included in this analysis: by randomized group

Characteristic	*N*	Overall *N* = 139	Amoxicillin/4 *N* = 26	Amoxicillin/8 *N* = 29	Co-amox/4 *N* = 29	Co-amox/8 *N* = 29	IVonly/5 *N* = 26	*p*-value^a^
Country	139							0.94
South Africa		57 (41%)	12 (46%)	12 (41%)	12 (41%)	11 (38%)	10 (38%)	
Uganda		30 (22%)	7 (27%)	6 (21%)	5 (17%)	7 (24%)	5 (19%)	
Zambia		25 (18%)	4 (15%)	5 (17%)	6 (21%)	6 (21%)	4 (15%)	
Zimbabwe		27 (19%)	3 (12%)	6 (21%)	6 (21%)	5 (17%)	7 (27%)	
Age (years)	139	1.0 (0.5, 1.7)	1.1 (0.7, 1.7)	1.0 (0.4, 1.4)	1.0 (0.6, 2.0)	0.9 (0.3, 1.4)	1.1 (0.6, 1.7)	0.6
Sex	139							0.6
Female		53 (38%)	8 (31%)	9 (31%)	14 (48%)	11 (38%)	11 (42%)	
Male		86 (62%)	18 (69%)	20 (69%)	15 (52%)	18 (62%)	15 (58%)	
Semi-quantitative point-of-care CRP (mg/L)	139							<0.001
10–40		26 (19%)	4 (15%)	9 (31%)	2 (6.9%)	4 (14%)	7 (27%)	
40–80		54 (39%)	12 (46%)	8 (28%)	13 (45%)	15 (52%)	6 (23%)	
>80		59 (42%)	10 (38%)	12 (41%)	14 (48%)	10 (34%)	13 (50%)	
Delivery mode	139							0.4
Caesarean section		34 (24%)	5 (19%)	8 (28%)	5 (17%)	6 (21%)	10 (38%)	
Vaginal		105 (76%)	21 (81%)	21 (72%)	24 (83%)	23 (79%)	16 (62%)	
MUAC (cm)	139	14.0 (13.0, 14.9)	14.0 (13.2, 15.5)	14.0 (12.5, 14.6)	14.0 (13.0, 14.5)	13.8 (12.4, 14.5)	14.2 (13.0, 14.9)	0.6
MUAC-for-age z-score	131^b^	−0.5 (−1.3, 0.2)	−0.5 (−1.1, 0.5)	−0.4 (−1.2, 0.2)	−0.5 (−1.6, 0.1)	−0.5 (−1.5, −0.3)	−0.6 (−1.2, 0.6)	>0.9
Oxygen supplementation at admission	139	119 (86%)	23 (88%)	24 (83%)	25 (86%)	25 (86%)	22 (85%)	>0.9
Antibiotic intake before admission (30 days)	139	14 (10%)	5 (19%)	3 (10%)	4 (14%)	1 (3.4%)	1 (3.8%)	0.3
Initial IV treatment	139							0.027
Ampicillin alone		4 (2.9%)	2 (7.7%)	2 (6.9%)	0 (0%)	0 (0%)	0 (0%)	
Ampicillin plus gentamicin		64 (46%)	11 (42%)	13 (45%)	15 (52%)	16 (55%)	9 (35%)	
Benzylpenicillin alone		7 (5.0%)	2 (7.7%)	0 (0%)	2 (6.9%)	2 (6.9%)	1 (3.8%)	
Benzylpenicillin plus gentamicin		29 (21%)	4 (15%)	6 (21%)	7 (24%)	4 (14%)	8 (31%)	
Cefotaxime alone		2 (1.4%)	1 (3.8%)	1 (3.4%)	0 (0%)	0 (0%)	0 (0%)	
Ceftriaxone alone		33 (24%)	6 (23%)	7 (24%)	5 (17%)	7 (24%)	8 (31%)	
Hospitalization length (days)^c^	139	4.7 (3.4, 6.0)	3.8 (2.8, 4.9)	4.4 (3.6, 6.0)	4.9 (2.7, 6.3)	4.5 (2.9, 6.8)	4.8 (4.7, 5.6)	0.3
Duration IV treatment at randomization (days)	139	0.7 (0.5, 0.9)	0.7 (0.5, 0.9)	0.7 (0.5, 0.8)	0.6 (0.4, 0.8)	0.7 (0.6, 0.9)	0.7 (0.5, 0.8)	0.3
Duration IV treatment at discharge (days)	139	3.0 (2.0, 5.0)	2.6 (2.0, 3.9)	2.7 (2.0, 4.0)	2.2 (1.8, 3.6)	3.0 (1.9, 5.8)	4.8 (4.6, 5.3)	<0.001
Duration oral treatment at discharge (days)	139	0.8 (0.0, 1.6)	0.8 (0.0, 1.6)	0.9 (0.8, 1.9)	0.7 (0.0, 1.3)	0.9 (0.8, 1.8)	0.0 (0.0, 0.0)	<0.001
Duration oral treatment at follow-up (days)	139	1.9 (0.0, 4.4)	1.3 (0.2, 2.2)	4.5 (3.3, 5.3)	1.5 (0.5, 2.2)	5.2 (3.0, 5.5)	0.0 (0.0, 0.0)	<0.001
Use of non-trial antibiotics	139	11 (7.9%)	4 (15%)	0 (0%)	2 (6.9%)	2 (6.9%)	3 (12%)	0.2
Days since last antibiotic intake at follow-up	139	21 (14, 24)	24 (9, 25)	20 (18, 22)	24 (18, 26)	21 (18, 21)	22 (6, 24)	0.034
Randomization sample available	139	95 (68%)	14 (54%)	14 (48%)	22 (76%)	23 (79%)	22 (85%)	0.009
Randomization and discharge sample available	139	60 (43%)	10 (38%)	10 (34%)	13 (45%)	11 (38%)	16 (62%)	0.3
Randomization and follow-up sample available	139	77 (55%)	11 (42%)	11 (38%)	18 (62%)	18 (62%)	19 (73%)	0.045
All three samples available	139	50 (36%)	8 (31%)	7 (24%)	11 (38%)	9 (31%)	15 (58%)	0.10

^a^Kruskal-Wallis test for continuous variables; for categorical variables Pearson’s Chi-squared test.

^b^z-score was missing from eight children aged <0.25 years old.

^c^Date of discharge was available, but not exact time; thus, midday was used.

showing n (%); Median (IQR).

### Gastrointestinal microbiota dynamics

**Fig. 2:**
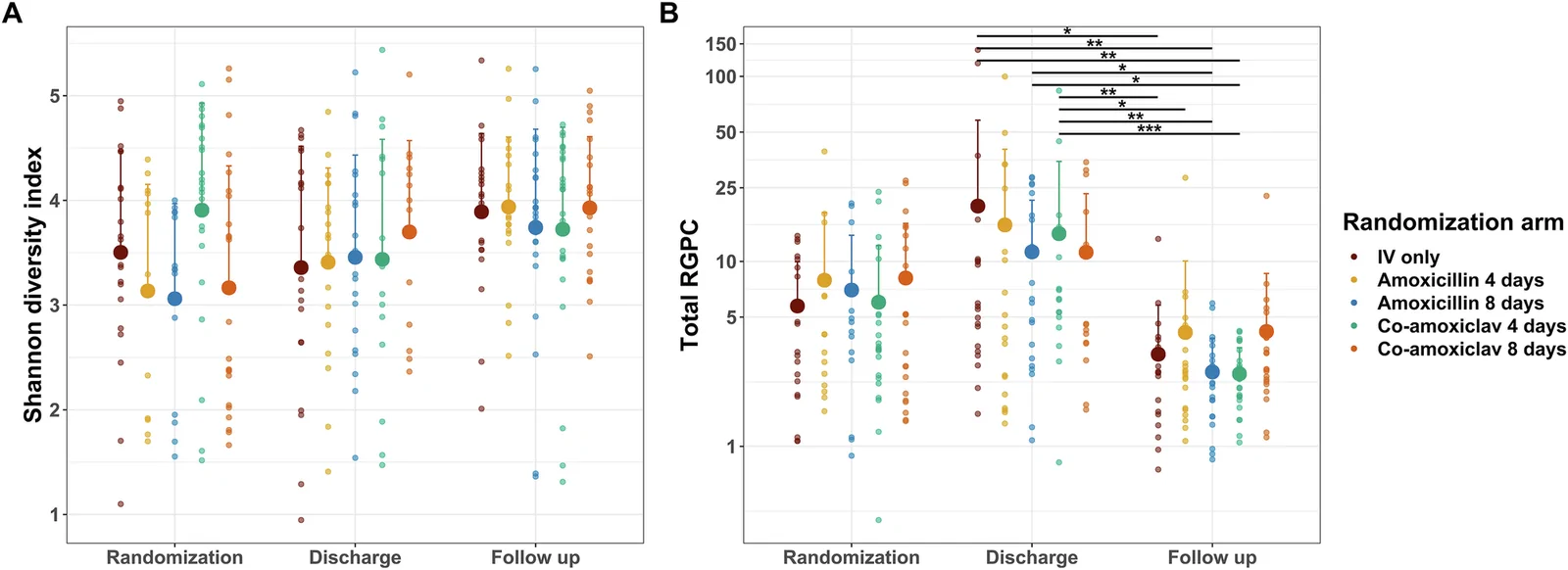
**A** Shannon alpha-diversity index per visit and randomized group. **B** Log-transformed total number of RGPCs per visit and randomized group. Comparisons were performed with pairwise Wilcoxon test, adjusting for 105 comparisons with the Bonferroni method. Lines and stars indicate differences between groups and timepoints with * adjusted *p*-value < 0.05, ** adjusted *p*-value < 0.01 and *** adjusted *p*-value < 0.001.

Shannon alpha-diversity index values were very heterogeneous across randomized groups and timepoints, although relatively stable between randomization to discharge, before increasing at follow-up (Fig. 2A). Of note, no significant differences were found between randomization groups within and across timepoints, although a trend towards higher diversity could be observed (Fig. 2A).

Multivariable linear regression models for Shannon alpha-diversity were constructed using backwards elimination (exit *p* = 0.1) on all factors in Table 1, forcing the structural design features “country” and “randomized group” into all models (plus “value at randomization” (baseline) for outcomes post-baseline). The only clinical factors independently associated with microbial community alpha-diversity at randomization were age and sex, with a marginal effect of country. Shannon alpha-diversity was higher in older children (adjusted *p*-value = 2.0 E-06), and lower in males (adjusted *p*-value = 0.045) (Table S2).

Of note, children had not necessarily completed their randomized oral antibiotics at discharge, but if well enough could be discharged home to complete their dispersible antibiotic tablet course. The largest independent contributor to changes in alpha-diversity at discharge compared to paired randomization samples (overall mean increase 0.04 (95% CI −0.26–0.34), unadjusted *p*-value = 0.80) was age (larger increases in older children: adjusted *p*-value = 0.013), with a marginal effect of antibiotic intake before admission (larger increases in those who had taken antibiotics before admission: adjusted *p*-value = 0.08) (Table S2). In contrast, the differences between paired follow-up and randomization samples (overall mean increase 0.35 (95% CI 0.11–0.59), unadjusted *p*-value = 0.004) were associated with age (larger increases in older children; adjusted *p*-value = 0.003), number of days since the last antibiotic exposure (larger increases in children who last took antibiotics longer ago; adjusted *p*-value = 0.011), and sex (smaller increases in males; adjusted *p*-value = 0.004) (Table S2). Notably, older children consistently had greater increases in diversity between timepoints.

**Fig. 3:**
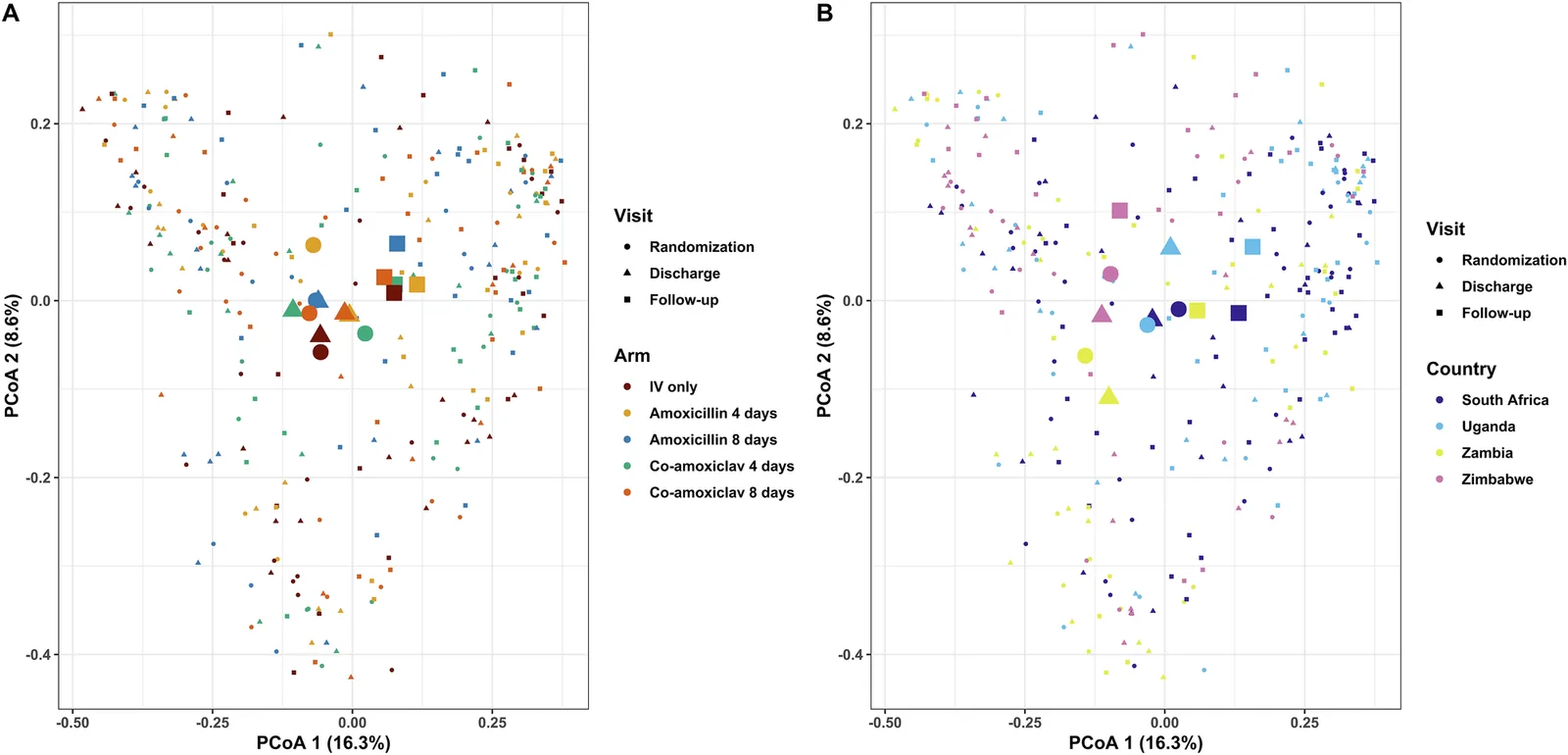
Principal component analysis performed on the community dissimilarity calculated with the Bray-Curtis index. Shape of the samples represent the timepoint when they were obtained, and the colour represents (**A**) randomized group and (**B**) country.

There were very small differences in microbial community dissimilarity between randomization and discharge across randomized groups, and the centroids tended to cluster, especially those at follow-up (Fig. 3A).

The contribution of each clinical factor to the microbial community at randomization was assessed with a distance-based redundancy analysis (db-RDA), which showed that country of origin (adjusted *p*-value = 0.002) and age (adjusted *p*-value = 0.001) had the strongest associations, while sex also showed a significant effect (adjusted *p*-value = 0.04) (Table S3). Indeed, when clustering the samples by country and timepoint, every country presented a distinct community at every timepoint (Fig. 3B). A beta regression analysis of the dissimilarity observed between randomization and discharge paired samples showed the largest association with sex (more similar in males; adjusted p-value = 0.0014), while MUAC-for-age z-score (more similar when z-scores were higher; adjusted *p*-value = 0.03), oxygen administration (more similar in those with oxygen supplementation at baseline; adjusted *p*-value = 0.07) and duration of oral treatment at discharge (higher dissimilarity in those that had undergone longer oral treatment before discharge: adjusted *p*-value = 0.07) showed marginal effects (Table S4). In contrast, dissimilarity between follow-up and randomization paired samples was mostly driven by age (more similar at older ages; adjusted *p*-value = 9.2 E-05) (Table S4).

### Gastrointestinal resistome dynamics

Antimicrobial resistance genes (ARGs) were detected with ARGs-OAP using the SARG database, and the tool returned normalized ARG counts per the estimated number of prokaryotic cells (RGPC)^9^. Comparing the total RGPC burden between timepoints, RGPCs tended to increase at discharge in all randomized groups, although again there was great heterogeneity between samples (Fig. 2B). At follow-up, RGPC numbers decreased to lower levels than at randomization for all groups, with significant differences between discharge and follow-up samples being observed between several randomization groups (Fig. 2B). Of note, all randomization groups followed these dynamics, without significant differences between them.

Excepting vancomycin, RGPCs for individual antibiotic classes were highly positively correlated with each other at discharge, with slightly higher correlations observed for multidrug RGPCs with other classes, such as polymyxin, aminoglycosides, sulphonamides or trimethoprim (correlations 0.09–0.68, mean 0.34) than between other individual antibiotic classes (excluding vancomycin: correlations −0.20–0.73, mean 0.25) (Fig. S2).

Multivariable linear regression models for the logarithmic transformation of total RGPC (approximately normally distributed) were constructed using backwards elimination (exit *p* = 0.1) on all factors in Table 1, forcing the structural design features “country” and “randomized group” into all models (plus “value at randomization” (baseline) for outcomes post-baseline). Total RGPC at randomization was lower in older children (adjusted *p*-value = 5.2 E-05), and was significantly higher in Ugandan children (versus South Africa, adjusted *p*-value = 0.010) (Table S2). The overall increases between paired randomization and discharge samples (overall mean increase 0.18 (95% CI 0.03–0.33), unadjusted *p*-value = 0.017) were most strongly associated with country (versus South Africa, greater increases in Uganda and smaller increases in Zimbabwe, adjusted *p*-value = 0.02) and age (smaller increases in older children, adjusted *p*-value = 0.011), with a marginal effect of sex (greater increases in males, adjusted *p*-value = 0.053) and use of non-trial antibiotics (smaller increases in those using non-trial antibiotics, adjusted *p*-value = 0.06) (Table S2). In contrast, RGPC decreased overall between randomization and follow-up (by mean −0.16 (95% CI −0.26 to −0.07), unadjusted p-value = 9.0 E-04), most strongly associated with country (smaller decreases in Uganda vs South Africa, adjusted *p*-value = 5.2 E-04), age (greater decreases in older children, adjusted *p*-value = 0.007) and duration of IV treatment (smaller decreases with longer IV durations, adjusted *p*-value = 0.03), with marginal effects of type of initial IV treatment (smaller decreases with benzylpenicillin alone versus ampicillin plus gentamicin, adjusted *p*-value = 0.09) and the duration of oral treatment (smaller decreases with longer oral durations, adjusted *p*-value = 0.09) (Table S2). Notably, age was negatively associated with both RGPC increases at discharge and RGPC decreases at follow-up, indicating that older children underwent smaller increases and then greater decreases in resistance gene abundance over time.

Following the total RGPC trend, beta-lactam RGPC numbers increased at discharge, although not significantly, and this increase was not observed for the IV only randomized group (Fig. S3A). At follow-up, the beta-lactam RGPC burden decreased to levels lower than at randomization, with high homogeneity between samples (Fig. S3A).

At discharge, beta-lactam RGPC abundance was positively associated with the abundance of Enterobacterales (correlation 0.55, *p*-value = 4.40 E-14), including abundance of *Enterobacter* spp. (correlation 0.57, *p*-value = 5.71 E-13), *Citrobacter* spp. (correlation 0.56, *p*-value = 5.47 E-12), *Klebsiella pneumoniae* (correlation 0.41, *p*-value = 1.47 E-07) and *Escherichia coli* (correlation 0.46, *p*-value = 3.00 E-10), but there was no evidence of association with the abundance of *Pseudomonas* spp. (correlation 0.07, *p*-value = 0.31) or *Acinetobacter* spp. (correlation −0.02, *p*-value = 0.78) (Fig. S2); these correlations were also observed when considering other timepoints. Of note, there was no evidence of association between the duration of IV (correlation 0.01, *p*-value = 0.87) or oral treatment (correlation −0.03, *p*-value = 0.71) before discharge and the abundance of beta-lactam RGPCs.

Vancomycin RGPC levels were low overall, with no evidence of change over time, and observed changes were most likely driven by natural variation (Fig. S3B).

### Differentially abundant species between groups

**Fig. 4:**
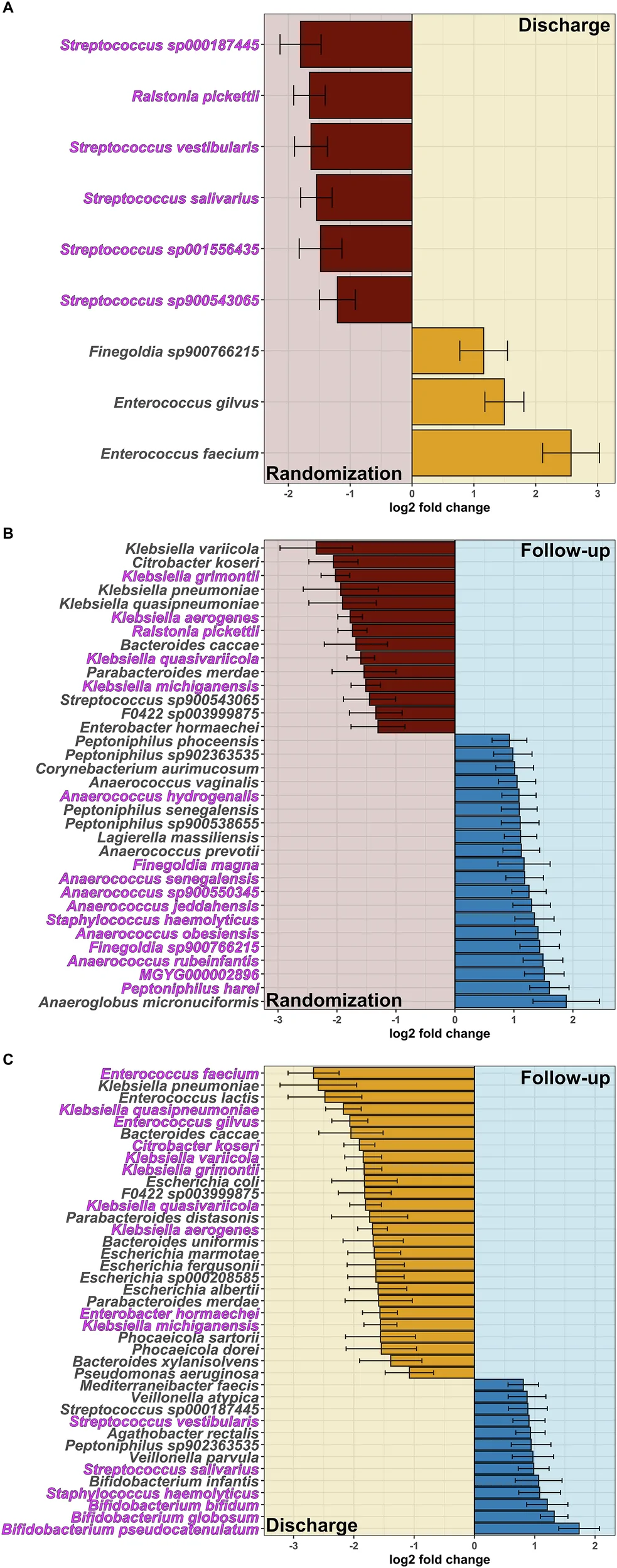
Differentially abundant species per visit, adjusting for country of origin, age and randomized group. Children had not necessarily completed their randomized total duration of antibiotics at discharge. Analysis was performed with DESeq2 using a false discovery rate cutoff of 0.1, ANCOM-BC2 with sensitivity analysis, and maaslin3 with an *q*-value cutoff of 0.1. Only species highlighted as significant by at least two methods and with a mean normalized number of 3000 reads or higher as calculated by DESeq2 were retained in the visualization. Coloured species were significant according to the three methods. Log fold change and standard error displayed per species is the closest to 0 among the three methods.

Following the trends observed in the PCoA, the intersection of DESeq2, maaslin3 and ANCOM-BC2 results showed few differentially abundant species between randomization and discharge after adjusting for country, age and randomized group, while differences between these two timepoints and follow-up were numerous (Fig. 4). Compared to an enrichment in streptococcal species at randomization, discharge samples were enriched in *Enterococcus* spp. (Fig. 4A). Follow-up samples showed higher abundance of *Finegoldia* spp., *Peptoniphilus* spp., *Anaerococcus* spp. *Staphylococcus haemolyticus* and *Bifidobacterium* spp. compared to both prior timepoints, while randomization and discharge samples were enriched in *Escherichia* spp., *Klebsiella* spp., *Bacteroides* spp. and *Enterococcus* spp. versus follow-up (Fig. 4B, C).

Analysing the differentially abundant bacterial species between randomized groups at discharge adjusting for age and country, no organisms showed differential abundance between groups, potentially due to little difference in IV and oral antibiotics received at discharge, with oral courses generally completed at home.

**Fig. 5:**
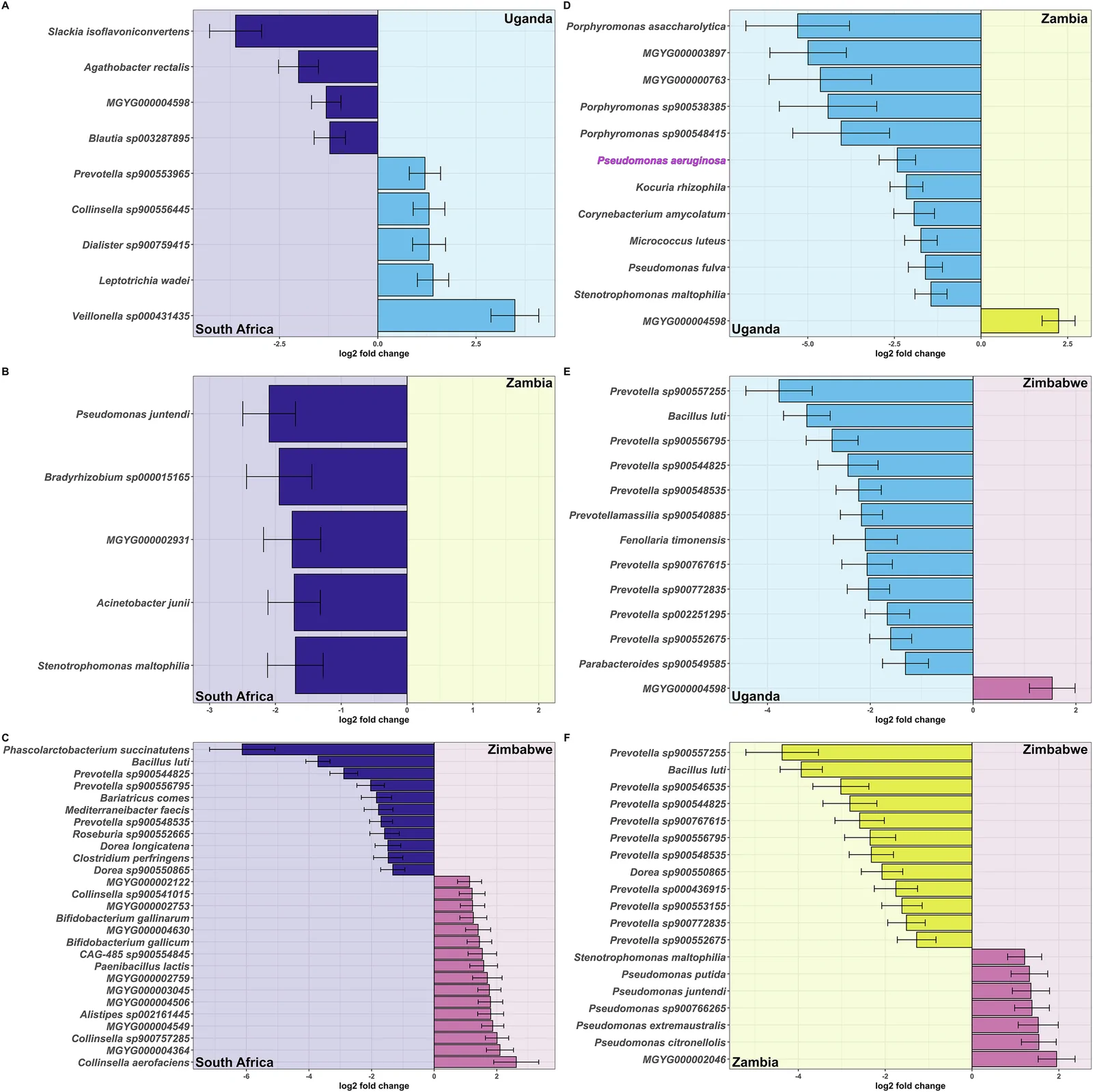
Differentially abundant species per country at follow-up, adjusted for age and randomized group. Analysis was performed with DESeq2 using a false discovery rate cutoff of 0.1, ANCOM-BC2 with sensitivity analysis, and maaslin3 with an *q*-value cutoff of 0.1. Only species highlighted as significant by at least two methods were retained in the visualization. Coloured species were significant according to the three methods. Log fold change and standard error displayed per species is the closest to 0 among the three methods.

As country had a large influence in the community composition, the analysis was also performed at randomization, adjusting for age, to determine bacterial species differentially abundant in samples from every country. Nevertheless, no significant differences were found at randomization between countries. Repeating the analysis at follow-up while adjusting for age and randomized group, some differences were found (Fig. 5). South African samples were enriched in *Agathobacter rectalis* and *Blautia* spp. compared to Ugandan samples (Fig. 5A), in *Stenotrophomonas maltophilia* compared to Zambian samples (Fig. 5B), and *Prevotella* spp. compared to Zimbabwean samples, which showed higher abundance of *Bifidobacterium* spp. and *Colinsella* spp. (Fig. 5C). Ugandan samples were enriched in *Porphyromonas* spp., *Pseudomonas aeruginosa* and *Stenotrophomonas maltophilia* compared to Zambian samples (Fig. 5D), and in *Prevotella* spp. compared to Zimbabwean samples (Fig. 5E). Finally, Zambian samples showed an enrichment in *Prevotella* spp. compared to Zimbabwean samples, which were enriched in *Pseudomonas* spp. and *Stenotrophomonas maltophilia* (Fig. 5F). All this points to an overall low abundance of *Prevotella* spp. in Zimbabwean samples and of *Stenotrophomonas maltophilia* in Zambian samples.

## Discussion

In this secondary sample analysis of the PediCAP trial, we describe the dynamics of the gastrointestinal microbiota and resistome of children with severe CAP randomized to different regimens of oral and IV antibiotics. These dynamics were most strongly associated with age and country, rather than initial IV treatment, and there was no evidence of an effect of randomization to step-down with narrower amoxicillin vs broader co-amoxiclav vs continuous IV, nor of total duration of antibiotic treatment. Despite increases in antimicrobial resistance gene abundance during hospitalization, four weeks later the resistance burden had decreased significantly, with a trend towards higher microbial diversity than at randomization.

Our study builds upon the clinical results from the main PediCAP trial analyses, where non-inferior cure rates were achieved by all step-down and duration groups compared to the current WHO treatment guidelines for 5 days IV^5^. Specifically, we highlight the lack of differential impact of the step-down approaches on the gastrointestinal microbiota, as well as of the initial IV treatment provided before and after randomization, with no significant impact on antimicrobial resistance development and no collateral impact on the microbiota of a strategy of stepping down to amoxicillin or co-amoxiclav from IV treatment when children were well enough to take oral medication. Of note, there was relatively little difference in total IV duration before discharge between the stepdown arms (2.2–3 days), meaning this cannot confound any relationship between randomised stepdown strategy and outcome, but children randomized to IV treatment alone had longer IV durations as expected (5 days). This could potentially be due to the use of the same antibiotic classes in all children, as initial IV treatments included ampicillin, ampicillin plus gentamicin, benzylpenicillin, benzylpenicillin plus gentamicin, cefotaxime or ceftriaxone, while step-down involved amoxicillin or co-amoxiclav, all beta-lactams with the same mechanism of action.

Overall, the abundance of RGPCs increased during hospitalization, with no evidence that this varied by randomized group. Oral administration can amplify ARG abundance in the gastrointestinal tract to a higher degree than IV administration^10^, and we found correlations between beta-lactam RGPC abundance and abundance of Enterobacterales, especially *Escherichia* and *Klebsiella*, which were higher at randomization and discharge. Indeed, many studies have reported an enrichment in gastrointestinal Enterobacterales after antibiotic treatment, very often linked directly to increases in beta-lactam ARG abundance^11–21^. This increased abundance of RGPC was, however, short-lived, and levels after four weeks were lower in all children, regardless of randomized group, accompanied by a trend to higher diversity. Many studies have also reported overall short times to recovery of the gastrointestinal microbiota after antibiotic treatment, often ranging from one week to several months, up to one year^13–16,22–25^. This suggests that the microbial community promptly recovers after hospital discharge and cessation of antibiotics to a healthier state, further supported by an enrichment in *Bifidobacterium* in the follow-up samples. Of note is that utilization of probiotics was not incorporated into the PediCAP trial design, so enrichment with *Bifidobacterium* likely represents acquisition of these species via the diet.

Colonization with vancomycin-resistant Enterococci (VRE) is a significant risk factor for nosocomial infections, potentially leading to severe complications^26^. However, vancomycin resistance genes were sparsely detected in our dataset and did not undergo great variations upon hospitalization.

The gastrointestinal microbiota during early-life stages is highly variable, although it follows common succession patterns towards higher diversity and lower ARG abundance, and the effect of factors like delivery mode, gestational age or early-life antibiotic treatment tends to dissipate over time^16,18–21,27–29^. Consistent with this, we found positive associations between age and microbial diversity, and negative associations between age and RGPC abundance. Additionally, age was positively associated with changes in diversity and negatively associated with changes in RGPC between timepoints, indicating a faster return to a diverse microbiota and a higher resilience to acquisition of resistance genes after antibiotic therapy in older children (noting that all children in the study were under 6 years, the median age was 1 year and the majority were under 2 years). It is possible that the lack of significant changes in diversity over time in our dataset is due to the sampling scheme besides a small sample size, as the randomization sample was obtained was obtained after IV antibiotics had already been started and there was a relatively short timeframe between randomization and discharge (most commonly 1–5 days).

Early-life microbiota and resistome dynamics are often understudied in low- and middle-income countries, especially involving diseases of public health significance in Africa^3,4,25^. A recent study has identified geographical differences in gastrointestinal resistome diversity and development^19^, and we have shown country of origin as one of the largest contributors to microbial community composition, much higher than antibiotic treatment regimen or duration. This highlights the need to expand microbiota studies to more diverse settings, especially considering the great variability in dietary patterns, lifestyle and environment, not only between African and Western populations, but also within Africa^4^.

Given that country of origin was one of the main variables influencing microbial community composition in this population and the lack of multi-country microbiome data in sub-Saharan Africa, we performed a differential abundance analysis between countries at randomization and follow-up. The microbial composition differences between countries included changes in abundance of potential pathogens and beneficial organisms. Previous studies have shown high abundance of *Bifidobacterium* in Zimbabwean populations in the first months of life, after which abundance is reduced due to replacement by other bacterial species, with no association with child growth and vaccine response^30,31^; however, in our dataset Zimbabwean samples had higher abundance of *Bifidobacterium* spp. than South Africa at follow-up. In South Africa, abundance of Proteobacteria has been described as higher in younger children and children presenting with infections, while *Prevotella* is overall one of the taxa with higher abundances^32–35^. In contrast, in our study Zimbabwean showed an overall low abundance of *Prevotella* compared to the rest of countries at follow-up, while high abundance of this genus is associated with diet in rural areas, although data regarding diet was not collected in this study^29^. These differences, while potentially not clinically relevant, emphasise the need for country-specific public health strategies to mitigate the development and spread of AMR^19^, supported by increased efforts to characterise the gastrointestinal microbiota in this population, and they might provide a base for future microbiome studies in African populations.

This study has several limitations. First, our samples were peri-rectal swabs, which may not accurately represent the gastrointestinal tract microbiota and resemble more the skin microbiota, although peri-rectal swabs show overall good correlation with stool samples regarding microbial community composition and detection of resistant organisms, except in critically ill patients^36–39^. This sample type was chosen as a compromise, considering the short timeframe between admission and discharge, as well as the interventions studied, as trying to collect stool samples could have resulted in delays, while rectal swabs were considered too invasive for this paediatric population. However, it may have contributed to the 27.4% loss of timepoints from analysis due to inadequate sequencing yield. Another limitation is the lack of additional samples between discharge and follow-up, which could potentially miss the maximum anticipated effect on diversity and ARG burden of oral step-down at the end of oral treatment, as well as the point when recovery of the microbial community starts. The study was relatively modest in size, limiting power, although still larger than many other studies in this field. As we were trying to estimate associations between clinical factors and outcomes in our sample, rather than generate a prediction model for use out-of-sample, we used backwards elimination rather than penalised regression approaches. Adjusting for uncertainty in the selection procedure would inflate p-values and confidence intervals, but would be highly unlikely to affect the strong (*p* < 0.001) associations we identified. Finally, data on variables known to influence the microbiome in infants, such as breastfeeding or introduction of solids in diet, was not collected, potentially overestimating the effect of surrogate variables like country in this study.

In conclusion, we have provided further support to the conclusions of the PediCAP trial, which favour early step-down and hospital discharge when possible. None of the oral step-down strategies or durations showed differential impact on the microbial community composition and antimicrobial resistance gene abundance compared to current guidelines. However, the differences in microbiota observed between countries involved in the study, especially regarding the presence of potential pathogens, highlight the need to expand current paediatric microbiota development studies to include more diverse populations.

## Methods

### Patient population

Children ≤6 years old admitted to hospital with severe CAP and judged to require at least 24 h of IV antibiotic treatment were enrolled into the PediCAP trial (ISRCTN63115131)^5^. Key exclusion criteria included semi-quantitative point-of-care C-reactive protein (CRP) levels <10 mg/L, hospitalization in the last 28 days, need for invasive ventilation or ICU admission, and long-term antibiotic prophylaxis. Upon admission, children received initial IV therapy following WHO CAP treatment guidelines; and were randomized within 24 h of initiating IV to continue IV antibiotics for five days, or step down to oral amoxicillin or oral co-amoxiclav (both formulated as dispersible tablets) when well enough for oral medication, the latter for a total treatment duration (IV+oral) of 4, 5, 6, 7, or 8 days (11 randomized groups).

Caregivers provided written informed consent for participation in the PediCAP trial and the current study, which involved further sampling and a follow-up visit, the latter being optional (Statistical Analysis Plan in Supplementary Information). As a secondary analysis of the PediCAP trial, this study adheres to the CONSORT guidelines of the main trial^5^. Ethical approval was obtained from local and national ethics committees and regulatory agencies in each recruiting country plus University College London, UK (University College London Research Ethics Committee, ref: 001,16423; University of Witwatersrand Human Research Ethics Committee, ref: 190913B; Makerere University School of Medicine Research Ethics Committee, ref: 2019-162; University of Zambia Biomedical Research Ethics Committee, ref: 328–2019; Joint Research Ethics Committee for the University of Zimbabwe, ref: 221/19). Written informed consent was provided by parents or legal guardians of participating children prior to any study procedures.

To maximise potential differences between groups, the analysis of these microbiology specimens was restricted to participants randomized to the IV only, amoxicillin step-down total 4 days, amoxicillin step-down 8 days, co-amoxiclav step-down 4 days, and co-amoxiclav step-down 8 days groups who had peri-rectal swabs obtained at randomization (baseline), discharge and follow-up (week 4).

Hospitalization length was calculated as Date of discharge – Date and time of admission; as exact discharge time was not available, time was set at midday. Duration of IV or oral treatment was calculated as Date and time of last reported IV or oral dose – Date and time of first reported IV or oral dose. Finally, days since last antibiotic at follow-up was calculated as Date of follow-up sample collection – Date of last reported antibiotic dose.

### Sample collection and processing

Peri-rectal samples were collected using DNA/RNA shield collection tubes with swab (Zymo Research), and were stored at −80 °C until transfer to the central laboratory at the University of Antwerp. DNA was extracted using the Fast DNA Spin Kit for Feces (MP Biomedicals) according to manufacturer’s instructions. Libraries were prepared using the Nextera XT Library Preparation Kit (Illumina) and sequenced in a NextSeq 500/550 instrument (Illumina) in a shotgun manner.

### Bioinformatic analysis

The peri-rectal shotgun sequencing data quality was assessed with FastQC v0.12.0, and reads were trimmed with TrimGalore v0.6.10, using a quality threshold of 30^40^. Host reads were removed with Hostile v2.0, using default settings^6^. Taxonomical classification of the microbial reads was performed using Kraken2 v2.1.3 with default settings and the UHGG (Unified Human Gastrointestinal Genome) database v2.0.2^7,41^. Additional species abundance estimation was performed with Bracken v2.5 on the Kraken2 output with the 150mers database^42^. Presence of contaminant species was determined using the decontam v1.30 R package, using as reference negative controls included in every DNA extraction run^8^. Species counts were normalized across samples using phyloseq v1.54 and seed 711. Alpha- (Shannon) and beta- (Bray-Curtis) diversity indices of the microbial community were obtained with the vegan v2.7.2 R package. AMR gene detection was performed with ARGs-OAP v3.2.4, using the SARG database v3.2.1, normalizing the antimicrobial resistance gene (ARG) counts per the estimated number of prokaryotic cells (RGPC)^9^.

### Statistical analysis

Normality of continuous variables was assessed with the Shapiro-Wilk test from base R, transforming in the case of gross violations. Associations between categorical variables were evaluated using Chi-squared tests, between categorical and continuous variables with the Kruskal-Wallis test from base R and between continuous variables with the Kendall correlation test. Differences between timepoints in continuous variables were assessed with pairwise Wilcoxon tests from base R, with Bonferroni adjustment. Multivariable linear regression models for Shannon alpha-diversity and log_10_-transformed RGPC (approximately normal) were constructed using the lm function from the Stats R package and backwards elimination (exit *p* = 0.1) on all factors in Table 1, forcing the structural design features “country”, “randomized group” (plus “value at randomization” (baseline) for outcomes post-baseline) into all models. Potential collinearity was assessed by first considering Spearman correlations between variables considered for inclusion (none >0.8) and comparing estimates from univariable and multivariable models. We refer to “marginal effects” when *p*-value of the variable was between 0.05 and 0.1. Community dissimilarity measured with the Bray-Curtis method was visualized with principal coordinate analysis (PCoA) plots, and the contribution of each clinical factor to this dissimilarity at randomization was assessed with distance-based redundancy analysis (db-RDA) using the capscale function from the vegan R package, while dissimilarity between timepoints was analysed by beta regression using the betareg function from the homonymous package v3.2.4. For models for randomization values, only patients providing samples at randomization were included in the analysis. Similarly, only patients that provided samples at both randomization and discharge, or randomization and 4-week follow-up, were included in the post-baseline models. Finally, DESeq2 v1.50.2, ANCOM-BC2 v2.12.0 and maaslin3 v1.1.2 were used to determine differentially abundant species between specific conditions using default settings, and only significant results provided by at least two tools were reported^43–45^.

## Supplementary information


Supplementary Information


## Data Availability

The datasets generated and analysed during the current study are available at ENA under bioproject number PRJEB90577 and at NCBI with bioproject number PRJNA1277853. Scripts are available at https://github.com/juanpablorod/PediCAP_analysis (https://doi.org/10.5281/zenodo.18404079).

## References

[1] GBD 2016 Lower Respiratory Infections Collaborators Estimates of the global, regional, and national morbidity, mortality, and aetiologies of lower respiratory infections in 195 countries, 1990–2016: a systematic analysis for the Global Burden of Disease Study 2016. *Lancet Infect. Dis.* **18**, 1191–1210 (2018). 30243584 10.1016/S1473-3099(18)30310-4 PMC6202443

[2] Wan, X. et al. Global burden of antimicrobial resistance in lower respiratory infections in 2021: *A systematic analysis*. **65**, 107431 (2025). 10.1016/j.ijantimicag.2024.107431 39734053

[3] Allali, I. et al. Human microbiota research in Africa: a systematic review reveals gaps and priorities for future research. *Microbiome* **9**, 241 (2021). 34911583 10.1186/s40168-021-01195-7 PMC8672519

[4] Pheeha, S. M., Tamuzi, J. L., Chale-Matsau, B., Manda, S. & Nyasulu, P. S. A Scoping Review Evaluating the Current State of Gut Microbiota Research in Africa. *Microorganisms* **11**, 10.3390/microorganisms11082118 (2023). PMC10458939 37630678

[5] Bielicki, J., Clements, M. & Musiime, V. Oral Step-Down, Optimal Drug and Total Duration of Antibiotic Treatment in African Children Hospitalised with Severe Community-Acquired Pneumonia: A Factorial Randomised Trial. *Lancet* (in press). 10.1016/S0140-6736(26)00879-2 42492562

[6] Constantinides, B., Hunt, M. & Crook, D. W. Hostile: accurate decontamination of microbial host sequences. *Bioinformatics* **39**. 10.1093/bioinformatics/btad728 (2023). PMC10749771 38039142

[7] Almeida, A. et al. A unified catalog of 204,938 reference genomes from the human gut microbiome. *Nat. Biotechnol.* **39**, 105–114 (2021). 32690973 10.1038/s41587-020-0603-3 PMC7801254

[8] Davis, N. M., Proctor, D. M., Holmes, S. P., Relman, D. A. & Callahan, B. J. Simple statistical identification and removal of contaminant sequences in marker-gene and metagenomics data. *Microbiome* **6**, 226 (2018). 30558668 10.1186/s40168-018-0605-2 PMC6298009

[9] Yin, X. et al. ARGs-OAP v2.0 with an expanded SARG database and Hidden Markov Models for enhancement characterization and quantification of antibiotic resistance genes in environmental metagenomes. *Bioinformatics* **34**, 2263–2270 (2018). 29408954 10.1093/bioinformatics/bty053

[10] Zhang, L., Huang, Y., Zhou, Y., Buckley, T. & Wang, H. H. Antibiotic administration routes significantly influence the levels of antibiotic resistance in gut microbiota. *Antimicrob. Agents Chemother.* **57**, 3659–3666 (2013). 23689712 10.1128/AAC.00670-13 PMC3719697

[11] Kelly, S. A. et al. Antibiotic Therapy and the Gut Microbiome: Investigating the Effect of Delivery Route on Gut Pathogens. *ACS Infect. Dis.* **7**, 1283–1296 (2021). 33843198 10.1021/acsinfecdis.1c00081 PMC7618120

[12] Wurm, J., Curtis, N. & Zimmermann, P. The effect of antibiotics on the intestinal microbiota in children - a systematic review. *Front. Allergy* **5**, 1458688 (2024). 39435363 10.3389/falgy.2024.1458688 PMC11491438

[13] MacPherson, C. W. et al. Gut Bacterial Microbiota and its Resistome Rapidly Recover to Basal State Levels after Short-term Amoxicillin-Clavulanic Acid Treatment in Healthy Adults. *Sci. Rep.* **8**, 11192 (2018). 30046129 10.1038/s41598-018-29229-5 PMC6060159

[14] Schwartz, D. J. et al. Effect of amoxicillin on the gut microbiome of children with severe acute malnutrition in Madarounfa, Niger: a retrospective metagenomic analysis of a placebo-controlled trial. *Lancet Microbe* **4**, e931–e942 (2023). 37866373 10.1016/S2666-5247(23)00213-6 PMC10620469

[15] McDonnell, L. et al. Association between antibiotics and gut microbiome dysbiosis in children: systematic review and meta-analysis. *Gut Microbes* **13**, 1–18 (2021). 33651651 10.1080/19490976.2020.1870402 PMC7928022

[16] Gibson, M. K., Crofts, T. S. & Dantas, G. Antibiotics and the developing infant gut microbiota and resistome. *Curr. Opin. Microbiol* **27**, 51–56 (2015). 26241507 10.1016/j.mib.2015.07.007 PMC4659777

[17] Bargheet, A. et al. Development of early life gut resistome and mobilome across gestational ages and microbiota-modifying treatments. *EBioMedicine* **92**, 104613 (2023). 37187112 10.1016/j.ebiom.2023.104613 PMC10192547

[18] Lebeaux, R. M. et al. The infant gut resistome is associated with E. coli and early-life exposures. *BMC Microbiol.* **21**, 201 (2021). 34215179 10.1186/s12866-021-02129-x PMC8252198

[19] Bargheet, A. et al. Dynamics of gut resistome and mobilome in early life: a meta-analysis. *EBioMedicine* **114**, 105630 (2025). 40048849 10.1016/j.ebiom.2025.105630 PMC11929092

[20] Zhao, L. et al. Temporal development and potential interactions between the gut microbiome and resistome in early childhood. *Microbiol Spectr.* **12**, e0317723 (2024). 38193687 10.1128/spectrum.03177-23 PMC10846076

[21] Patangia, D. V. et al. Early life exposure of infants to benzylpenicillin and gentamicin is associated with a persistent amplification of the gut resistome. *Microbiome* **12**, 19 (2024). 38310316 10.1186/s40168-023-01732-6 PMC10837951

[22] Panda, S. et al. Short-term effect of antibiotics on human gut microbiota. *PLoS One* **9**, e95476 (2014). 24748167 10.1371/journal.pone.0095476 PMC3991704

[23] Lu, S., Huang, Q., Wei, B. & Chen, Y. Effects of beta-Lactam Antibiotics on Gut Microbiota Colonization and Metabolites in Late Preterm Infants. *Curr. Microbiol* **77**, 3888–3896 (2020). 32970172 10.1007/s00284-020-02198-7

[24] Reyman, M. et al. Effects of early-life antibiotics on the developing infant gut microbiome and resistome: a randomized trial. *Nat. Commun.* **13**, 893 (2022). 35173154 10.1038/s41467-022-28525-z PMC8850541

[25] Luchen, C. C. et al. Impact of antibiotics on gut microbiome composition and resistome in the first years of life in low- to middle-income countries: A systematic review. *PLoS Med.* **20**, e1004235 (2023). 37368871 10.1371/journal.pmed.1004235 PMC10298773

[26] Flokas, M. E., Karageorgos, S. A., Detsis, M., Alevizakos, M. & Mylonakis, E. Vancomycin-resistant enterococci colonisation, risk factors and risk for infection among hospitalised paediatric patients: a systematic review and meta-analysis. *Int J. Antimicrob. Agents* **49**, 565–572 (2017). 28336313 10.1016/j.ijantimicag.2017.01.008

[27] Gasparrini, A. J. et al. Persistent metagenomic signatures of early-life hospitalization and antibiotic treatment in the infant gut microbiota and resistome. *Nat. Microbiol.* **4**, 2285–2297 (2019). 31501537 10.1038/s41564-019-0550-2 PMC6879825

[28] Rodriguez, J. M. et al. The composition of the gut microbiota throughout life, with an emphasis on early life. *Micro. Ecol. Health Dis.* **26**, 26050 (2015). 10.3402/mehd.v26.26050 PMC4315782 25651996

[29] Iddrisu, I. et al. Malnutrition and Gut Microbiota in Children. *Nutrients* **13**, 10.3390/nu13082727 (2021). PMC8401185 34444887

[30] Zhou, D. T., Mudhluli, T. E., Hall, L. J., Manasa, J. & Munyati, S. A Scoping Review of Gut Microbiome and Bifidobacterium Research in Zimbabwe: Implications for Future Studies. *Pediatr. Health Med. Ther.* **14**, 483–496 (2023). 10.2147/PHMT.S414766 PMC10743709 38145055

[31] Robertson, R. C. et al. The gut microbiome and early-life growth in a population with high prevalence of stunting. *Nat. Commun.* **14**, 654 (2023). 36788215 10.1038/s41467-023-36135-6 PMC9929340

[32] Nel Van Zyl, K. et al. Association between clinical and environmental factors and the gut microbiota profiles in young South African children. *Sci. Rep.* **11**, 15895 (2021). 34354176 10.1038/s41598-021-95409-5 PMC8342602

[33] Mahdavinia, M. et al. Atopic dermatitis and food sensitization in South African toddlers: Role of fiber and gut microbiota. *Ann. Allergy Asthma Immunol.* **118**, 742–743 e743 (2017). 28583264 10.1016/j.anai.2017.04.011

[34] Goosen, C. et al. The effect of oral iron supplementation on the gut microbiota, gut inflammation, and iron status in iron-depleted South African school-age children with virally suppressed HIV and without HIV. *Eur. J. Nutr.* **61**, 2067–2078 (2022). 34997267 10.1007/s00394-021-02793-9

[35] Krishnamoorthy, S., Coetzee, V., Kruger, J., Potgieter, H. & Buys, E. M. Dysbiosis Signatures of Fecal Microbiota in South African Infants with Respiratory, Gastrointestinal, and Other Diseases. *J. Pediatr.* **218**, 106–113 e103 (2020). 31952848 10.1016/j.jpeds.2019.11.029

[36] Turner, G. et al. Rectal swabs are a reliable method of assessing the colonic microbiome. *Int. J. Med. Microbiol* **312**, 151549 (2022). 35114582 10.1016/j.ijmm.2022.151549

[37] Short, M. I. et al. Comparison of rectal swab, glove tip, and participant-collected stool techniques for gut microbiome sampling. *BMC Microbiol.* **21**, 26 (2021). 33446094 10.1186/s12866-020-02080-3 PMC7809826

[38] Fair, K. et al. Rectal Swabs from Critically Ill Patients Provide Discordant Representations of the Gut Microbiome Compared to Stool Samples. *mSphere* **4**, 10.1128/mSphere.00358-19 (2019). PMC6656869 31341070

[39] Kubiak, J. M. et al. Comparison between Perianal Swab and Stool Specimens for Detecting Colonization with Extended-Spectrum Beta-Lactamase-Producing and Fluoroquinolone-Resistant Enterobacterales. *J. Clin. Microbiol* **60**, e0023422 (2022). 35695506 10.1128/jcm.00234-22 PMC9297816

[40] Andrews, S. (2010).

[41] Wood, D. E., Lu, J. & Langmead, B. Improved metagenomic analysis with Kraken 2. *Genome Biol.* **20**, 257 (2019). 31779668 10.1186/s13059-019-1891-0 PMC6883579

[42] Lu, J., Breitwieser, F. P., Thielen, P. & Salzberg, S. L. Bracken: estimating species abundance in metagenomics data. *Peerj Comput. Sci*. ARTN e104 10.7717/peerj-cs.104 (2017). 10.7717/peerj-cs.104 PMC12016282 40271438

[43] Love, M. I., Huber, W. & Anders, S. Moderated estimation of fold change and dispersion for RNA-seq data with DESeq2. *Genome Biol.* **15**, 550 (2014). 25516281 10.1186/s13059-014-0550-8 PMC4302049

[44] Nickols, W. A. et al. MaAsLin 3: Refining and extending generalized multivariable linear models for meta-omic association discovery. *bioRxiv* 10.1101/2024.12.13.628459 (2024). 41540124 PMC12982127

[45] Lin, H. & Peddada, S. D. Multigroup analysis of compositions of microbiomes with covariate adjustments and repeated measures. *Nat. Methods* **21**, 83–91 (2024). 38158428 10.1038/s41592-023-02092-7 PMC10776411

