# Supplementary material for: Impact of co-amoxiclav versus amoxicillin on the gastrointestinal microbiota in sub-Saharan children hospitalized with pneumonia
Source: NPJ Antimicrob Resist. 2026 May 22;4:54. doi: 10.1038/s44259-026-00216-5 (PMC13530132; doi:10.1038/s44259-026-00216-5)
Supplement: Supplementary file 1 [file 44259_2026_216_MOESM1_ESM.pdf]

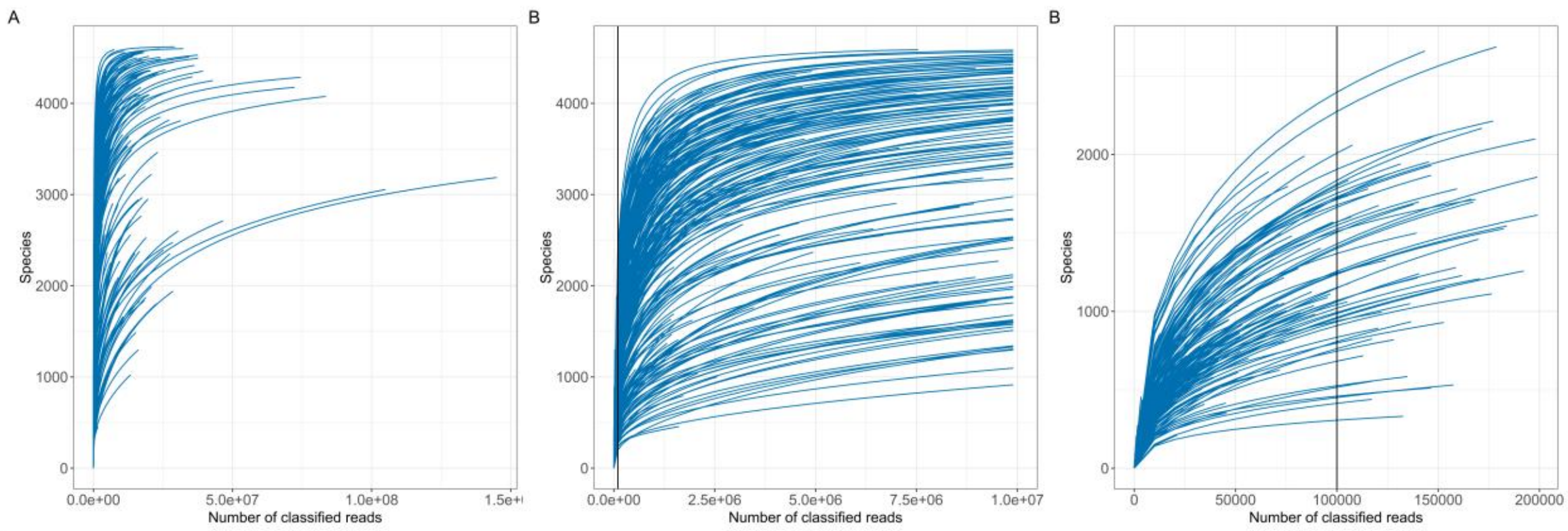

Figure S1. Microbial species rarefaction curves for (A) all samples, (B) all samples with x-axis limited to 1,000,000 classified reads and (C) samples with < 200,000 classified reads. (B) and (C) include a vertical line drawn at 100,000 reads.

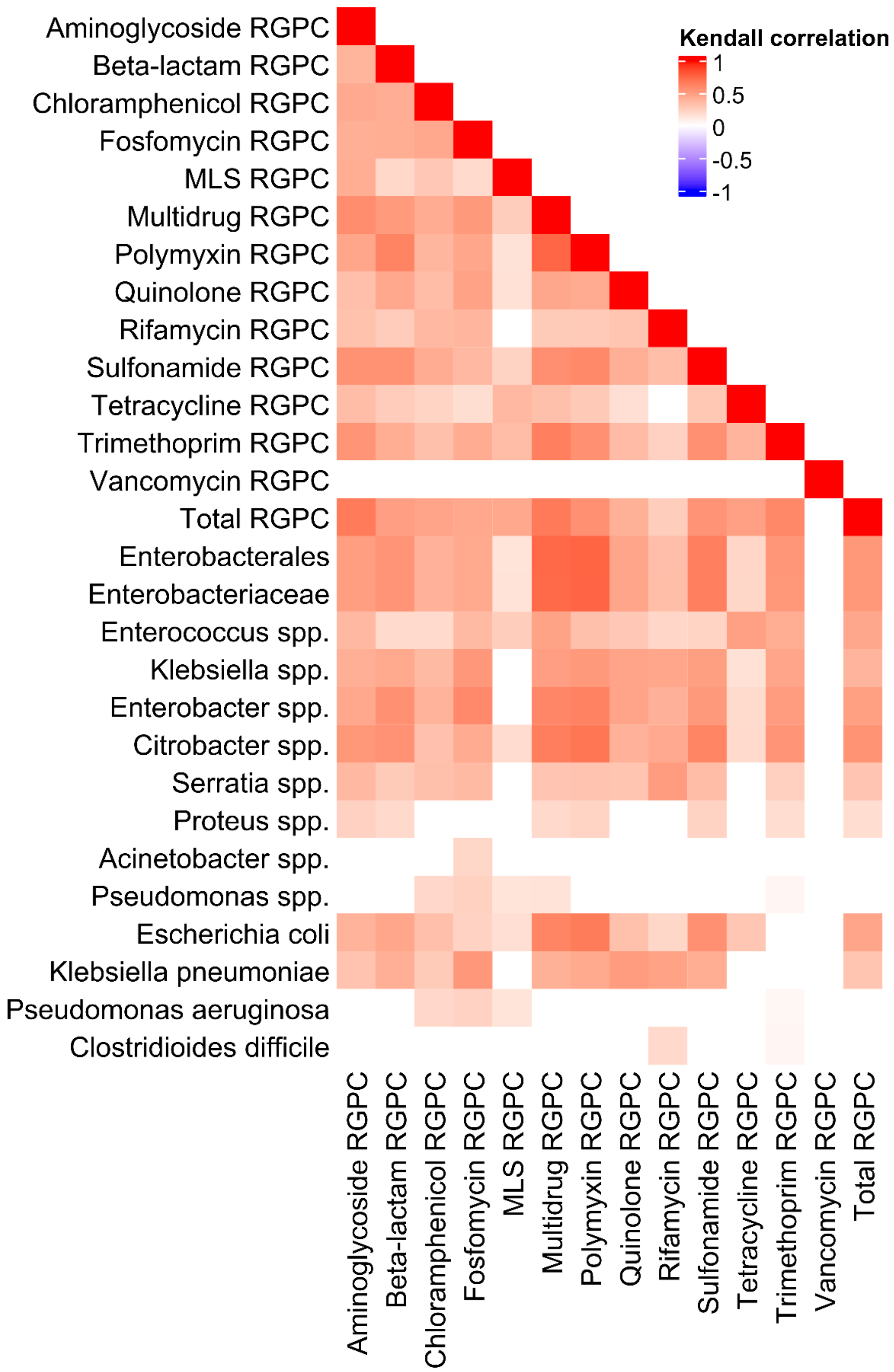

Figure S2. Correlation matrix for abundance of antibiotic class-specific RGPCs and a selection of organisms described in the statistical analysis plan at discharge visit. MLS denotes macrolide-lincosamide-streptogramin.

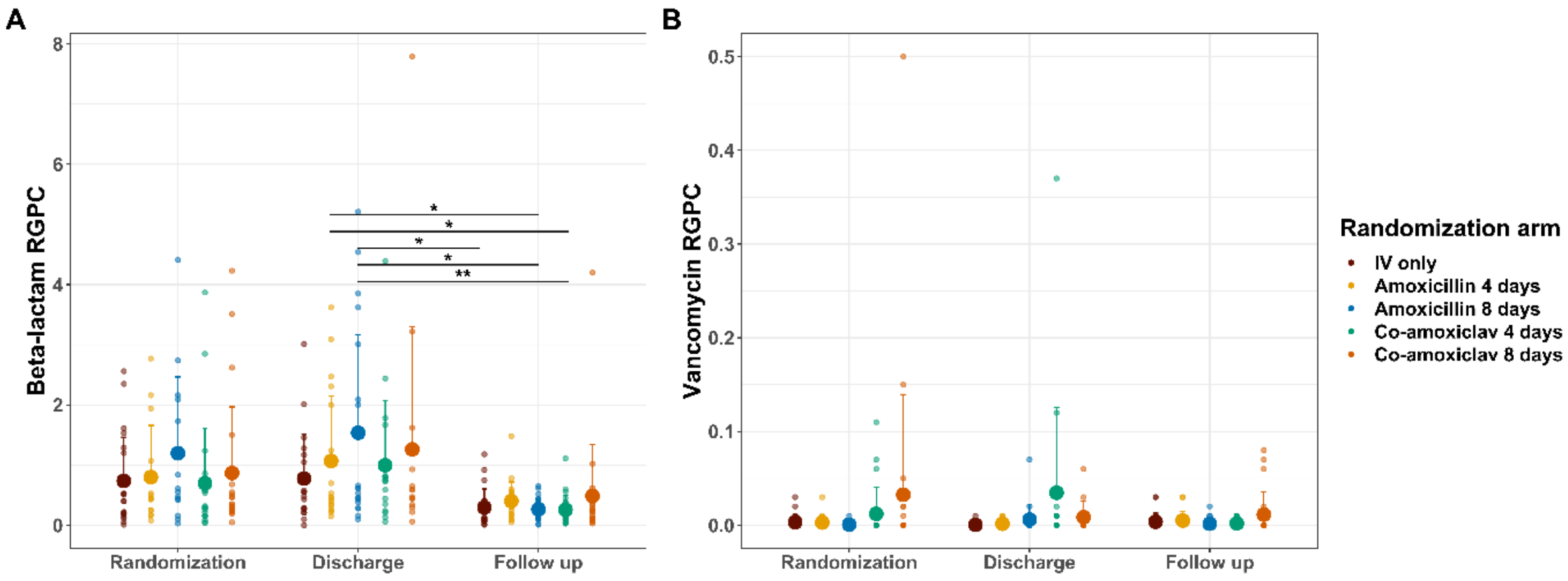

Figure S3. (A) Beta-lactam RGPC per visit and randomized group. (B) Vancomycin RGPCs per visit and randomized group. Comparisons were performed with pairwise Wilcoxon test, adjusting for 105 comparisons with the Bonferroni method. Lines and stars indicate differences between groups and timepoints with * adjusted p-value <0.05 and ** adjusted p-value <0.01.

Table S1. Clinical characteristics associated with sub-study patients included in this analysis: by country

Characteristic	N	Overall N = 139	South Africa N = 57	Uganda N = 30	Zambia N = 25	Zimbabwe N = 27	p-value[1]
**Randomized group**	139						0.94
Amoxicillin/4		26 (19%)	12 (21%)	7 (23%)	4 (16%)	3 (11%)	
Amoxicillin/8		29 (21%)	12 (21%)	6 (20%)	5 (20%)	6 (22%)	
Co-amox/4		29 (21%)	12 (21%)	5 (17%)	6 (24%)	6 (22%)	
Co-amox/8		29 (21%)	11 (19%)	7 (23%)	6 (24%)	5 (19%)	
IV only/5		26 (19%)	10 (18%)	5 (17%)	4 (16%)	7 (26%)	
**Age (years)**	139	1.0 (0.5, 1.7)	1.0 (0.6, 1.5)	1.3 (0.7, 2.1)	1.1 (0.6, 1.6)	0.6 (0.3, 1.3)	0.054
**Sex**	139						0.3
Female		53 (38%)	22 (39%)	10 (33%)	7 (28%)	14 (52%)	
Male		86 (62%)	35 (61%)	20 (67%)	18 (72%)	13 (48%)	
**Semi-quantitative point-of-care CRP (mg/L)**	139						<0.001
10-40		26 (19%)	16 (28%)	9 (30%)	1 (4.0%)	0 (0%)	
40-80		54 (39%)	22 (39%)	14 (47%)	8 (32%)	10 (37%)	
>80		59 (42%)	19 (33%)	7 (23%)	16 (64%)	17 (63%)	
**Delivery mode**	139						0.004
Caesarean section		34 (24%)	23 (40%)	4 (13%)	3 (12%)	4 (15%)	
Vaginal		105 (76%)	34 (60%)	26 (87%)	22 (88%)	23 (85%)	
**MUAC (cm)**	139	14.0 (13.0, 14.9)	14.0 (13.0, 15.1)	14.3 (13.6, 15.0)	13.0 (12.3, 14.2)	13.2 (12.4, 14.8)	0.010
**MUAC-for-age z-score**	131[2]	-0.5 (-1.3, 0.2)	-0.4 (-1.1, 0.6)	-0.4 (-0.8, 0.2)	-0.9 (-2.1, -0.3)	-0.5 (-1.2, 0.2)	0.2
**Oxygen supplementation at admission**	139	119 (86%)	52 (91%)	18 (60%)	24 (96%)	25 (93%)	<0.001
**Antibiotic intake before admission (30 days)**	139	14 (10%)	2 (3.5%)	2 (6.7%)	6 (24%)	4 (15%)	0.023
**Initial IV treatment**	139						<0.001
Ampicillin alone		4 (2.9%)	4 (7.0%)	0 (0%)	0 (0%)	0 (0%)	
Ampicillin plus gentamicin		64 (46%)	35 (61%)	28 (93%)	0 (0%)	1 (3.7%)	
Benzylpenicillin alone		7 (5.0%)	0 (0%)	0 (0%)	7 (28%)	0 (0%)	
Benzylpenicillin plus gentamicin		29 (21%)	0 (0%)	0 (0%)	4 (16%)	25 (93%)	
Cefotaxime alone		2 (1.4%)	1 (1.8%)	1 (3.3%)	0 (0%)	0 (0%)	
Ceftriaxone alone		33 (24%)	17 (30%)	1 (3.3%)	14 (56%)	1 (3.7%)	
**Hospitalization length (days)**[3]	139	4.7 (3.4, 6.0)	3.7 (2.6, 4.9)	3.9 (3.2, 4.8)	7.6 (5.6, 9.1)	5.0 (4.5, 6.8)	<0.001
**Duration IV treatment at randomization (days)**	139	0.7 (0.5, 0.9)	0.7 (0.5, 0.8)	0.7 (0.5, 0.8)	0.6 (0.2, 0.8)	0.8 (0.6, 0.9)	0.049
**Duration IV treatment at discharge (days)**	139	3.0 (2.0, 5.0)	2.0 (1.7, 3.6)	3.8 (2.7, 4.8)	5.6 (3.0, 7.8)	4.5 (2.6, 5.3)	<0.001
**Duration oral treatment at discharge (days)**	139	0.8 (0.0, 1.6)	1.2 (0.0, 1.8)	0.8 (0.0, 0.9)	0.8 (0.0, 1.8)	0.8 (0.0, 1.8)	0.072
**Duration oral treatment at follow-up (days)**	139	1.9 (0.0, 4.4)	2.2 (1.2, 5.2)	1.5 (0.0, 3.4)	0.8 (0.0, 4.3)	1.8 (0.0, 4.5)	0.2
**Use of non-trial antibiotics**	139	11 (7.9%)	2 (3.5%)	0 (0%)	6 (24%)	3 (11%)	0.004
**Days since last antibiotic intake at follow-up**	139	21 (14, 24)	22 (19, 25)	21 (9, 23)	23 (17, 24)	20 (13, 23)	0.4
**Randomization sample available**	139	95 (68%)	36 (63%)	20 (67%)	20 (80%)	19 (70%)	0.5
**Randomization and discharge sample available**	139	60 (43%)	18 (32%)	15 (50%)	15 (60%)	12 (44%)	0.086
**Randomization and follow-up sample available**	139	77 (55%)	25 (44%)	18 (60%)	18 (72%)	16 (59%)	0.10
**All three samples available**	139	50 (36%)	13 (23%)	14 (47%)	13 (52%)	10 (37%)	0.035

[1] Kruskal-Wallis test for continuous variables; for categorical variables Pearson's Chi-squared test.
[2] z-score was missing from eight children aged <0.25 years old
[3] Date of discharge was available, but not exact time; thus, midday was used
Note: showing n (%); Median (IQR)

Table S2. Linear regression models and ANOVA results obtained after backwards elimination for Shannon and log_{10}RGPC at randomization, paired difference of values at discharge and randomization, and paired difference of values at follow-up and randomization. The structural design features "country", "randomized group" and "randomization value" were forced into all models, if applicable for the timepoint. ANOVA test results are only shown for factors with multiple degrees of freedom in the main model.

**Shannon alpha-diversity at randomization**					
Model formula	Shannon randomization ~ Country + Age + Sex				
Reference	Country: South Africa, Sex: Female				
Coefficients	Estimate	95% CI		p-value	Sig
(Intercept)	3.3	2.8	3.7	<2 E-16	***
Country: Uganda	-0.3	-0.8	0.2	0.2	
Country: Zambia	-0.1	-0.6	0.4	0.6	
Country: Zimbabwe	-0.4	-0.9	0.2	0.2	
Age	0.4	0.3	0.6	2.0 E-06	***
Sex: Male	-0.4	-0.8	-0.01	0.045	*
ANOVA					
Response: Shannon randomization	Degree of freedom	Sum of Square	F value	p-value	Sig
Country	3	6.3	2.6	0.06	.

**log_{10}RGPC at randomization**					
Model formula	log_{10}RGPC randomization ~ Country + Age				
Reference	Country: South Africa				
Coefficients	Estimate	95% CI		p-value	Sig
(Intercept)	0.7	0.6	0.9	6.4 E-15	***
Country: Uganda	0.3	0.1	0.5	0.013	*
Country: Zambia	0.01	-0.2	0.2	0.9	
Country: Zimbabwe	0.2	-0.04	0.4	0.1	
Age	-0.1	-0.2	-0.1	5.2 E-05	***
ANOVA					
Response: log_{10}RGPC randomization	Degree of freedom	Sum of Square	F value	p-value	Sig
Country	3	1.5	4.0	0.010	*

**Discharge - randomization Shannon alpha-diversity**					
Model formula	Shannon discharge - randomization ~ Country + Shannon randomization + IV only + Drug + Duration + Age + Antibiotic intake before admission				
Reference	Country: South Africa, IV only: No, Drug: Amoxicillin, Duration: 4, Antibiotic intake before admission: No				
Coefficients	Estimate	95% CI		p-value	Sig
(Intercept)	1.7	0.9	3.2	0.02	*
Country: Uganda	-0.1	-0.7	0.6	0.9	
Country: Zambia	0.1	-0.6	0.8	0.7	
Country: Zimbabwe	-0.4	-1.2	0.3	0.3	
Shannon randomization	-0.8	-1.2	-0.5	1.2 E-06	***
IV only: Yes	0.1	-0.6	0.8	0.8	
Drug: Co-amoxiclav 7:1	0.3	-0.3	1.0	0.3	
Duration: 8	0.1	-0.2	0.9	0.3	
Age	0.4	0.1	0.7	0.013	*

Antibiotic intake before admission: Yes	0.8	-0.1	1.6	0.08	.
ANOVA					
Response: Shannon discharge - randomization	Degree of freedom	Sum of Square	F value	p-value	Sig
Country	3	2.4	1.0	0.4	

**Discharge - randomization log_{10}RGPC**					
Model formula	log_{10}RGPC discharge - randomization ~ Country + log_{10}RGPC randomization + IV only + Drug + Duration + Age + Sex + Use of non-trial antibiotic				
Reference	Country: South Africa, IV only: No, Drug: Amoxicillin, Duration: 4, Sex: Female, Use of non-trial antibiotic: No				
Coefficients	Estimate	95% CI		p-value	Sig
(Intercept)	1.1	0.5	1.5	0.0016	**
Country: Uganda	0.2	-0.2	0.5	0.4	
Country: Zambia	-0.03	-0.4	0.3	0.9	
Country: Zimbabwe	-0.2	-0.7	0.2	0.3	
log_{10}RGPC randomization	-0.9	-1.3	-0.5	1.3 E-04	***
IV only: Yes	0.1	-0.3	0.4	0.7	
Drug: Co-amoxiclav 7:1	-0.1	-0.4	0.2	0.5	
Duration: 8	-0.02	-0.4	0.2	0.6	
Age	-0.2	-0.3	-0.1	0.011	*
Sex: Male	0.3	-0.004	0.6	0.053	.
Use of non-trial antibiotic: Yes	-0.4	-0.8	0.02	0.06	.
ANOVA					
Response: log_{10}RGPC discharge - randomization	Degree of freedom	Sum of Square	F value	p-value	Sig
Country	3	2.4	3.4	0.02	*

**Follow-up - randomization Shannon alpha diversity**					
Model formula	Shannon follow-up - randomization ~ Country + Shannon randomization + IV only + Drug + Duration + Age + Sex + Days since last antibiotic at follow-up				
Reference	Country: South Africa, IV only: No, Drug: Amoxicillin, Duration: 4, Sex: Female				
Coefficients	Estimate	95% CI		p-value	Sig
(Intercept)	2.2	1.6	3.5	4.5 E-04	***
Country: Uganda	-0.1	-0.6	0.3	0.5	
Country: Zambia	0.1	-0.3	0.5	0.7	
Country: Zimbabwe	-0.6	-1.1	-0.1	0.013	*
Shannon randomization	-0.9	-1.0	-0.7	1.5 E-14	***
IV only: Yes	0.1	-0.4	0.5	0.8	
Drug: Co-amoxiclav 7:1	0.1	-0.3	0.4	0.8	
Duration: 8	0.4	0.01	0.7	0.04	*
Age	0.2	0.1	0.4	0.003	**
Sex: Male	-0.5	-0.8	-0.2	0.004	**
Days since last antibiotic at follow-up	0.03	0.01	0.05	0.011	*
ANOVA					
Response: Shannon follow-up - randomization	Degree of freedom	Sum of Square	F value	p-value	Sig
Country	3	1.0	0.8	0.5	

**Follow-up - randomization log_{10}RGPC**	
Model formula	log_{10}RGPC follow-up - randomization ~ Country + log_{10}RGPC randomization + IV only + Drug + Duration + Age + Initial IV treatment + Duration IV treatment + Duration oral treatment
Reference	Country: South Africa, IV only: No, Drug: Amoxicillin, Duration: 4, Initial IV treatment: Ampicillin plus gentamicin

Coefficients	Estimate	95% CI		p-value	Sig
(Intercept)	0.2	-0.1	0.4	0.2	
Country: Uganda	0.3	0.2	0.5	3.0 E-04	***
Country: Zambia	-0.01	-0.3	0.2	0.9	
Country: Zimbabwe	0.01	-0.3	0.3	0.96	
log_{10}RGPC randomization	-1.0	-1.1	-0.8	1.2 E-15	***
IV only: Yes	0.003	-0.2	0.2	0.97	
Drug: Co-amoxiclav 7:1	0.1	-0.004	0.3	0.06	.
Duration: 8	-0.001	-0.1	0.1	0.9	
Age	-0.1	-0.1	-0.02	0.007	**
Initial IV treatment: benzylpenicillin alone	0.4	0.04	0.7	0.03	*
Initial IV treatment: benzylpenicillin plus gentamicin	0.1	-0.2	0.4	0.7	
Initial IV treatment: ceftriaxone alone	0.2	-0.03	0.4	0.09	.
Duration IV treatment	0.03	0.003	0.05	0.03	*
Duration oral treatment	0.02	-0.002	0.03	0.09	.
ANOVA					
Response: log_{10}RGPC follow-up - randomization	Degree of freedom	Sum of Square	F value	p-value	Sig
Country	3	1.1	6.7	5.2 E-04	***
Initial IV treatment	3	0.4	2.2	0.09	.

Table S3. Distance-based redundancy analysis (dbRDA) model obtained after backwards elimination for dissimilarity of the microbial communities at randomization. The structural design feature "country" was forced into the models.

Dissimilarity at randomization					
Model formula	Species Bray-Curtis dissimilarity randomization ~ Country + Age + Sex				
Variable	Degree of freedom	Sum of Square	F value	p-value	Sig
Country	3	2.0	1.8	0.002	**
Age	1	1.9	5.1	0.001	***
Sex	1	0.6	1.6	0.04	*
Residual	90	33.7			

Table S4. Beta regression models and Wald test results obtained after backwards elimination for dissimilarity of the microbial communities at discharge and randomization, and follow-up and randomization. The structural design features "country" and "randomized group" were forced into all models. Wald test results are only shown for factors with multiple degrees of freedom in the main model.

**Discharge - randomization dissimilarity**					
Model formula	Species Bray-Curtis dissimilarity discharge - randomization ~ Country + IV only + Drug + Duration + Sex + MUAC-for-age z-score + Oxygen supplementation + Duration oral treatment at discharge				
Reference	Country: South Africa, IV only: No, Drug: Amoxicillin, Duration: 4, Sex: Female, Oxygen supplementation: No				
Coefficients	Estimate	95% CI		p-value	Sig
(Intercept)	1.4	0.5	2.3	0.002	**
Country: Uganda	0.2	-0.4	0.9	0.5	
Country: Zambia	0.4	-0.2	1.0	0.2	
Country: Zimbabwe	0.2	-0.5	0.9	0.6	
IV only: Yes	0.8	0.1	1.5	0.02	*
Drug: Co-amoxiclav 7:1	0.2	-0.4	0.8	0.5	
Duration: 8	-0.2	-0.8	0.3	0.5	
Sex: Male	-0.8	-1.3	-0.3	0.0014	**
MUAC-for-age z-score	-0.3	-0.5	-0.03	0.03	*
Oxygen supplementation: Yes	-0.6	-1.2	0.1	0.07	.
Duration oral treatment at discharge	0.2	-0.01	0.4	0.07	.
Wald test					
Variable	Degree of freedom		F value	p-value	Sig
Country	3		0.53	0.67	

**Follow-up - randomization dissimilarity**					
Model formula	Species Bray-Curtis dissimilarity follow-up - randomization ~ Country + IV only + Drug + Duration + Age				
Reference	Country: South Africa, IV only: No, Drug: Amoxicillin, Duration: 4				
Coefficients	Estimate	95% CI		p-value	Sig
(Intercept)	1.4	0.7	1.9	0.003	**
Country: Uganda	0.6	0.1	1.2	0.01	*
Country: Zambia	0.4	-0.1	0.9	0.14	
Country: Zimbabwe	0.5	-0.1	1.1	0.10	
IV only: Yes	-0.1	-0.6	0.5	0.8	
Drug: Co-amoxiclav 7:1	0.1	-0.4	0.5	0.7	
Duration: 8	-0.01	-0.5	0.4	0.9	
Age	-0.3	-0.5	-0.2	9.2 E-05	***
Wald test					
Variable	Degree of freedom		F value	p-value	Sig
Country	3		2.2	0.09	.

Supplementary information: Members of the PediCAP network

Participating Sites

Mozambique:

Universidade Eduardo Mondlane, Faculdade de Medicina, Maputo: Cláudia Massitela, Winete Joaquim, Alice Maieca, Uneisse Cassia, Quim Adriano, Elias Manjate.

Hospital Geral de Mavalane, Maputo: Nádia Manjate, Mara Bombe.

Hospital Provincial da Matola, Matola: Eleutéria Macanze, Sheila Nhacumba.

Hospital Geral José Macamo, Maputo: Faiaz Issa, Natércia Florindo.

Hospital Central de Maputo, Maputo: Sonia Martins, Ana Sigaúque, Rosalina Chilengue.

South Africa:

Africa Health Research Institute, Durban: Nigel Klein, Leatitia Kampiire, Anne Derache, Samkelisiwe Buthelezi, Senamile Makari.

Chris Hani Baragwanath Academic Hospital, University of the Witwatersrand, Johannesburg: Zainab Waggie, Ziyaad Dangor, Sandisiwe Nkosi, Sihle Zindela, Nomhle Nkumana, Ntombi Khumalo, Lethabo Nkosi, Tshepiso Msibi, Brandon Jones, Lora Frank.

Zambia:

University Teaching Hospital Zambia, Lusaka: Monica Kapasa, Gae Mundundu, Patricia Ngoma, Jacqueline H. Kaira, Oliver Mwenechanya, Anne Chimolula, Bwalya Simunyola, Martha Sakala, Joyce C. Lungu, Chimuka Kwale, Dorothy Zangata, Naomi Mumba.

Chawama General Hospital Zambia, Lusaka: Jacqueline Mulako, Mundimba Makwamba, Frederick Chansa, Carolyn Zulu.

Zimbabwe:

University of Zimbabwe Faculty of Medicine and Health Sciences, Clinical Research Centre, Harare: Kusum J. Nathoo, Ennie Chidziva, Shepherd Mudzingwa, Misheck Phiri, Ruth Nhema, Godfrey Musoro, Vivian Mumbiro, Moses Chitsamatanga, Joy Chimhanzi, Shirley Mutsai, Dorcas Murungu, Wendy Mapfumo.

Trial coordination and conduct

Laboratory of Medical Microbiology, University of Antwerp, Antwerp, Belgium: Juan Pablo Rodriguez Ruiz, Tomislav Kostyanev, Christine Lammens, Servaas Hiel, Sofie Van Mieghem, Gert Leten, Liesbeth Bryssinck.

Fondazione Penta, Padova, Italy: Musakanya Ching'andu, Francesca Viero.

University of Cape Town, Cape Town, South Africa: Susan Cleary, Lucy Cunnama, Mutsawashe Chitando, Paolo Denti, Roeland Wasmann, Manna Semere Gebreyesus, Jennie van Dyk.

Swiss Tropical and Public Health Institute, Basel, Switzerland: Günther Fink, Gillian Levine.

Global Antibiotic Research and Development Partnership, Geneva, Switzerland: Sally Ellis.

Centre for Neonatal and Paediatric Infection, St George's, University of London, UK: Kamla Pillay, Louise Hill, Tatiana Munera-Huertas, Jan Goelen.

Medical Research Council Clinical Trials Unit at University College London, London, UK: Clare Shakeshaft, Farjana Haque, Hanh Nguyen, Emily Dennis, Tatiana Sarfati, Brendan Murphy, Kolpona Begum, Nishdha Naufal, Hannah Sweeney, Jemima Shickle, Fatima Mohamed, Lee Barker, Marion Hall, Mallory Williams, Charlotte Hartley, Annabelle South.

Trial oversight

Trial Steering Committee members: Elizabeth Molyneux (Chair), Elizabeth Obimbo, Shamim Qazi, Somwe Wa Somwe, Robin Green.

Data Monitoring Committee members: Mainga Hamaluba, Tim Peto (Chair), Andrew Prendergast, Haroon Saloojee, Margaret Siwale.

Endpoint Review Committee members: Felicity Fitzgerald, Christina Obiero, Anna Turkova (Chair).

Supplementary information: Statistical analysis plan

1 Introduction

The overall aim of the PediCAP trial was to evaluate the impact of oral step-down to amoxicillin or co-amoxiclav and of duration of antibiotic therapy on effectiveness, safety and selection of antibiotic resistance in severe childhood community-acquired pneumonia (CAP) in Africa. This would result in the development of an evidence-base for recommending oral step-down antibiotic treatment and future choice of drug, dose, duration and delivery formulation that achieves resolution of symptoms of severe CAP while minimising the acquisition of resistant bacteria.

2 Objectives

2.1 PediCAP main trial objectives

The overall objectives of the main PediCAP trial (protocol Section 1.5) were to determine:

- Is the rate of clinical cure superior with co-amoxiclav 7:1 versus amoxicillin oral step-down therapy? (co-primary objective)
- What is the optimal antibiotic treatment duration that achieves good rates of clinical cure whilst minimising length of hospital stay, toxicity and acquisition of multidrug antimicrobial resistance? (co-primary objective)
- Does this optimal duration vary by key characteristics, such as age, underlying conditions or risk factors such as HIV exposure, malnutrition or severity, suggesting that antibiotic selection or duration should be personalised to specific subgroups? (secondary objective)

2.2 Microbiology sub-study objectives

As stated in protocol Section 9.2.3, the original microbiology sub-study objectives were to determine, in a subset of children, the impact of randomisation to co-amoxiclav 7:1 versus amoxicillin oral step-down therapy, and to different durations of total antibiotic therapy, on

- Changes in nasopharyngeal carriage of antibiotic-resistant (e.g. pencillin non-susceptible, methicillin-resistant) Gram positive bacteria
- Changes in faecal carriage of extended spectrum beta-lactamases (ESBL)-producing Gram negative bacteria

Whilst the primary objective of the microbiology sub-study remains to compare randomised groups, the most efficient methods for assaying antimicrobial resistance (AMR) across a range of species are now molecular rather than culture-based. Further, compared to when the protocol was written, the relative importance of the gut microbiome over the nasal microbiome has increased. Therefore, the primary objective of the microbiology sub-study is now to compare between randomised groups

- Changes in the total burden of AMR in faecal samples from metagenomic shotgun sequencing, normalised based on predicted number of bacterial cells detected using prokaryotic marker genes from baseline to discharge and to day 28

Secondary objectives are to compare between randomised groups

- Changes in the total burden of ESBL genes and vancomycin-resistant Enterococcus (VRE) in faecal samples from baseline to discharge and to day 28
- Changes in the microbiome in faecal samples from metagenomic shotgun sequencing, normalised based on sequencing depth from baseline to discharge and to day 28

For both these objectives, the hypotheses are that shorter total antibiotic treatment, and step-down to a less broad spectrum oral antibiotic (amoxicillin rather than co-amoxiclav), will be associated with smaller initial changes to the gut microbiome and resistome, with an earlier reversion to baseline.

Additional secondary objectives are

- To identify baseline factors associated with characteristics of the baseline faecal microbiome (primarily AMR burden as above, but also species diversity), including site, age, sex, etc.
- To identify the co-carriage and/or co-detection of 45 RNA viruses and bacteria potentially involved in CAP pathogenesis in baseline nasopharyngeal samples using the ThermoFisher (TF) CARD multiplex assay.
- To identify the serotypes of any *Streptococcus pneumoniae* identified in baseline nasopharyngeal samples using Nanopore sequence based meta-serotyping.

3 Microbiology sub-study outcomes

Raw faecal fastq files undergo quality trimming with TrimGalore (quality threshold 30) and host read removal with Hostile using default settings. For the processed faecal sequencing files, the outcomes are:

- Total number of resistance genes (based on mapping with >80% identity to the SARG database) normalized per estimated number of prokaryotic cells (RGPC) determined using ARGs-OAP with default settings and database [primary].
- Total number of ESBL resistance genes detected and normalized as above.
- Alpha- (Shannon and Chao) and beta-diversity (Bray-Curtis) indices of the microbial community.
- Proportion of reads mapping to specific organisms (*Enterococcus* spp., Enterobacterales, *Escherichia coli*, *Klebsiella pneumoniae*, *Klebsiella* spp, *Enterobacter* spp., *Citrobacter* spp, *Serratia* spp. *Proteus* spp., *Acinetobacter* spp., *Pseudomonas* aeruginosa, *Pseudomonas* spp., *Clostridiodes difficile*) determined by kraken2 and bracken with default settings and the UHGG v2.3 database.

For the TF multiplex CARD assay, the outcomes are

- Presence of each of the 45 pathogens included in the assay,
- Co-detection rates of the pathogens included in the assay.

For the pneumococcal serotyping, the outcome is

- Pneumococcal serotypes detected.
- Penicillin-binding protein variants detected.

- Other streptococci detected based on the *lytA* gene sequence.

4 Trial design

PediCAP was a multi-arm, open label randomised controlled trial of treatment strategies (drug and duration) for severe pneumonia in children.

All children initiated treatment with intravenous antibiotics as per the relevant site's standard of care within predefined regimens. Then, children were effectively firstly randomised 5:5:1 to either oral step-down amoxicillin (amoxicillin group); oral step-down co-amoxiclav (co-amoxiclav group); or to remain on intravenous antibiotics (IV group). Within the amoxicillin and co-amoxiclav groups, children were simultaneously further randomised 1:1:1:1:1 to receive 4, 5, 6, 7 or 8 days of total antibiotic treatment (from the start of intravenous antibiotic therapy). Children randomised to the IV group received 5 days of treatment with one of the PediCAP standard of care IV antibiotic regimens. Thus, each child in the main trial was randomised to one of 11 different groups. Oral treatment commenced when the child was judged well enough to take oral antibiotics, and were given with food where possible.

Treatment was be open-label without blinding and was dispensed at the point of oral step-down for the amoxicillin and co-amoxiclav groups, for the duration needed to complete the randomised total antibiotic course (starting at the time intravenous antibiotics were administered, excluding any antibiotics taking in the community prior to admission).

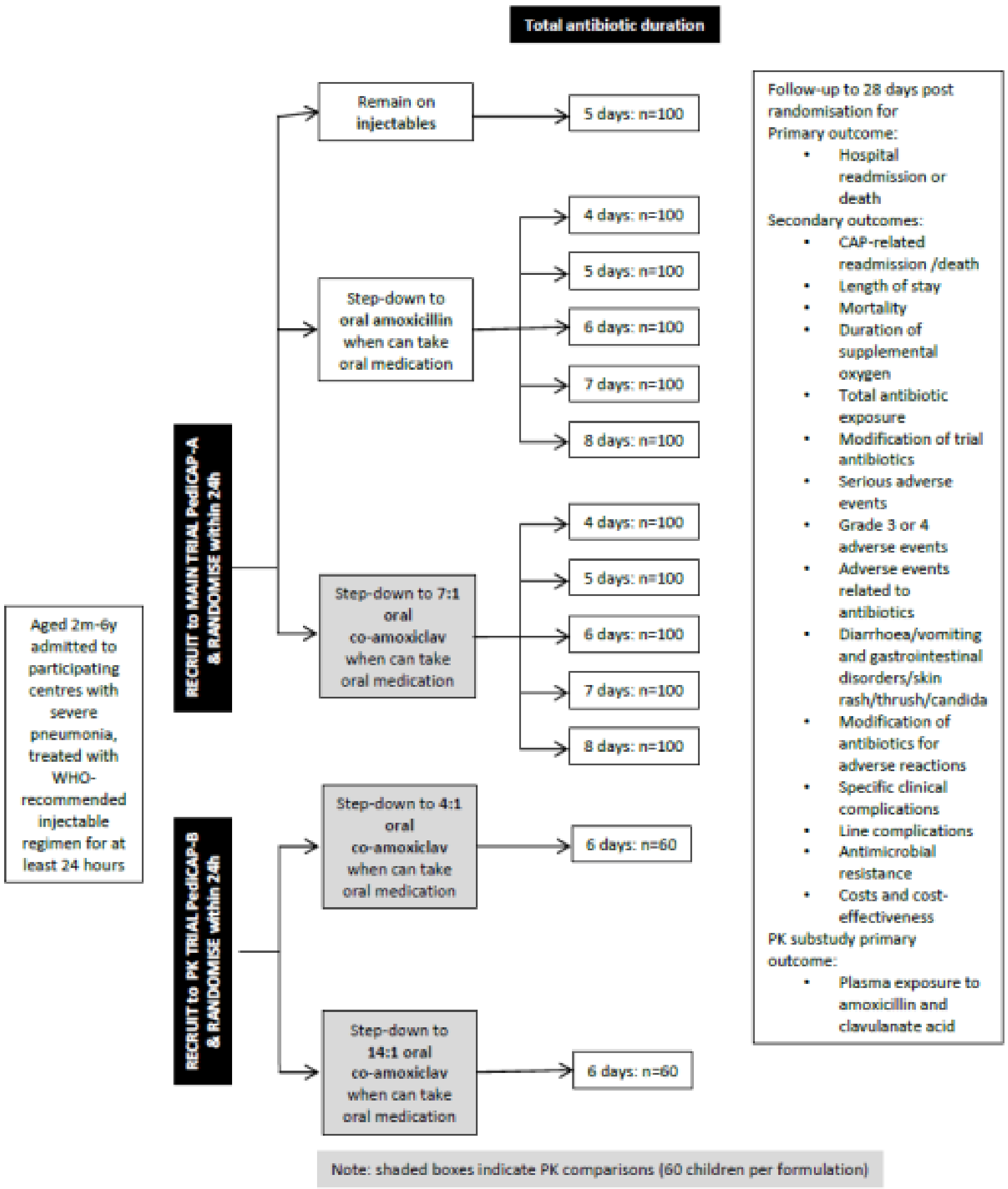

5 Trial Population

5.1 Inclusion criteria

- Aged 2 months to 6 years inclusive.
- Weighing >= 3kg and <30kg.
- Admitted to hospital with severe pneumonia judged to require at least 24h of intravenous antibiotics by the treating physician.
- Difficulty breathing (with or without cough reported by parent/carer) PLUS one or more of:
    - Central cyanosis or hypoxaemia (room air pulse oximetry <90%).
    - Any sign of severe respiratory distress (e.g. severe chest indrawing, grunting, nasal flaring, head nodding).
    - Signs of pneumonia (fast breathing (defined as respiratory rate ≥50 breaths per minute at age 2-11 months and ≥40 breaths per minute at age 1 years or older) or chest indrawing) PLUS a general danger sign (inability to breastfeed or drink, lethargy or reduced level of consciousness, convulsions, moderate/severe malnutrition).

- About to initiate or already initiated intravenous benzylpenicillin plus gentamicin, ampicillin plus gentamicin, benzylpenicillin or ampicillin alone, ceftriaxone alone or cefotaxime alone.
- Received at most 24h of these intravenous antibiotics at the point of randomisation (that is, first dose of any intravenous antibiotics must have been administered no more than 24h previously at randomisation).
- Parent/carer willing to accept and adhere to all possible randomised allocations for their child (including 5 days of intravenous antibiotics if joining PediCAP-A) and signed written informed consent available from parent/carer.
- Available for follow-up for the entire study period; specifically, parent/carer willing to return with their child to clinic at 4 weeks, and be contacted at minimum by telephone at weeks 1, 2 and 3.

5.2 Exclusion criteria

- Point-of-care semi-quantitative C-reactive protein (CRP) test < 10mg/l at screening (very unlikely to represent severe pneumonia requiring antibiotics).
- Likely nosocomial pneumonia (onset >48h post-admission).
- Admitted to hospital overnight in the last 28 days (possibility of nosocomially-acquired pneumonia).
- Known or anticipated need for invasive ventilation or admission to intensive care.
- Clinician considers this episode to be predominantly due to reactive airways disease (e.g. asthma) (wheeze responsive to bronchodilators).
- Clinician considers this episode to be due to viral bronchiolitis alone in a child under 1 year.
- Documented allergy to any drug from the penicillin class or contra-indications to penicillin/amoxicillin/co-amoxiclav.
- Anticipated need for systemic treatment with an antibiotic other than trial regimens during hospital admission or in the following 28 days (e.g. for *Pneumocystis jiroveci*).
- On long-term antibiotics for prophylaxis or treatment (e.g. for tuberculosis treatment or cotrimoxazole prophylaxis for HIV infection).
- Previously enrolled in PediCAP.

5.3 Microbiology study

A subset of 330 children participating in the main trial were recruited into the microbiology study if additional optional consent was provided.

6 Microbiological Sampling

Patients participating in the microbiology study will provide:

- Nasopharyngeal swab: each patient was sampled on Day 1 (day of inclusion) and either at either discharge (which could be before completion of the total antibiotic course) or week 4, determined by an additional randomization. Randomisation between discharge and week 4 for the second nasopharyngeal swab ensures that changes in nasal flora could be assessed

unbiasedly at both timepoints, without requiring three swabs per child, as they tend to be invasive for the patient.

- Peri-rectal swab or stool: each patient was sampled on Day 1 (day of inclusion), at discharge (which could be before completion of the total antibiotic course) and at week 4.

The samples were stored at -80°C for batched transfer to the University of Antwerp for analysis. Following analysis, the samples are stored in a biobank at the University of Antwerp central laboratory in a pseudonymized manner.

7 Microbiology analysis populations

The primary analyses will be based on complete cases for each outcome defined in Section 3 above, including all participants consenting to the microbiology study, providing the relevant samples and with calculable outcome data.

Samples were not shipped or analysed but destroyed for any patients withdrawing consent for future use of samples in the microbiology study.

Peri-rectal samples showing less than 100,000 classified reads based on mapping to the UHGG database with kraken and bracken will not be included in the analysis.

8 Analysis methods

Each outcome will be analysed using the appropriate generalised linear model, specifically

- Normalised resistance gene burden and other continuous outcomes which are not restricted to a range: normal linear regression, potentially after transformation for approximate normality
- Proportions (restricted to the range 0-1): beta regression
- Binary outcomes: logistic regression.

All models (other than those for baseline data only) will adjust for the randomised group and randomisation stratification factors (following the main trial Statistical Analysis Plan), specifically including as a factor amoxicillin (reference) vs co-amoxiclav vs IV, total randomised duration of treatment (reference category 5 days) (in separate models as a factor and as a linear term), and site.

Additional baseline factors will be considered on top of these models (see Section 9).

Methods to analyse change from baseline need to adjust for baseline values. Initially a simpler model will be fitted restricted to participants with data at baseline and each subsequent timepoint, adjusting for the observed baseline value, essentially restricting to complete cases for both baseline and subsequent timepoints. However, missing data is expected to be around 30% due to low read numbers. We will therefore compare this analysis just adjusting for randomised group and stratification factors with a more efficient method using random effects models, since these can include all data whether or not participants have missing data at baseline or subsequently, providing unbiased estimates of change from baseline. In these models, each timepoint is included as an outcome, and participant-level random effects are included for each timepoint with an unstructured correlation between them. Randomised group is included as a partial interaction, since there is no association between randomised group and baseline by definition. Stratification factors are included

as a full interaction since they may affect baseline and subsequent timepoints, with potentially different associations at each timepoint which could affect estimates of randomised group. Note the goal is not to estimate interactions but to adjust for them, with the primary focus on the estimates of randomised groups on change from baseline. If inference regarding the effect of randomised groups is similar from the two approaches, the simpler approach will be used to investigate the impact of other factors.

A key challenge with the design is that some participants randomised to longer antibiotic durations will have been discharged before completing their total course: that is, the sample taken at discharge and assayed for this substudy will not reflect the total antibiotic exposure. Further other participants may have received antibiotics for longer than randomised. Analyses using antibiotics received will be subject to confounding bias; analyses based on the randomisation will be free from confounding bias but will be subject to dilution bias in terms of estimating the direct effect of antibiotics received. Firstly, the primary analysis based on intention-to-treat (as randomised) will be interpreted in the context of the percentages of children who had stepped down as planned (including timing of step-down) or switched antibiotics (oral or IV) which will be summarised for the population of children included in the final models. Secondly, we will attempt to adjust for this in two ways.

- Per-protocol: We will first use a per-protocol analysis restricting to participants who received their assigned total duration of treatment ±1 day, and who only received allocated antibiotics or WHO-permitted IV regimens. This comprises only 66% of children and hence is a potentially biased comparison.
- As treated: Instead of using randomised group, we will use a categorical indicator for whether the child had stepped down to oral amoxicillin, oral co-amoxiclav or only received IVs at the time of the sample, and for the total duration of antibiotics actually received.
- We will also combine both approaches.

9 Other covariates in the microbiology study

The covariates that might influence the microbiology endpoints and will be investigated for their effects on the outcomes above include:

- Age: Younger children (<2 years old) present a more unstable microbiome, which might affect observed longitudinal changes.
- Mode of delivery: It has been reported that the delivery mode impacts the microbiome in the early stages of life.
- Medical history: The following conditions/procedures might have an influence in the gut and nasopharyngeal microbial communities: asthma, chronic lung disease, PCV vaccination.
- Previous antibiotic intake: Depending on the class of antibiotic, there might be effects of prior antibiotic intake (specific antibiotic not necessarily recorded) and initial IV regimen (recorded but only taken for <24h before baseline).
- Other concurrent medication: Use of laxatives or anti-acids might impact the microbial community in the gastrointestinal tract.

- Measures of malnutrition based on weight for age, height for age, BMI for age and muac for age.
- We will not formally consider HIV status as factor, because exclusion criteria relating to long-term antibiotic use meant very few HIV-infected children were enrolled (1%). However, it will be considered as a specific reason which could explain for example presence/absence of specific co-infections from TF card assays or multiple serotypes on meta-serotyping.

Depending on the results of the ITT and as-treated analysis, further exploratory analyses of specific antibiotics actually received (e.g. including changes in IV or other antibiotics and time of step-down) may be undertaken.

10 Phased approach

As metagenomic sequencing is an expensive assay, for efficiency samples at baseline, discharge and day 28 will first be assayed for participants in the microbiology sub-study randomised to 4 or 8 days total antibiotics including oral step-down, or 5 days' IV. Each run will contain samples from all randomised groups. An analysis of these data using the methods described above (treating total antibiotic duration as a categorical factor) will be used to assess the value of conducting additional assays in those randomised to 5, 6 or 7 days total antibiotic duration. This decision will be taken by the microbiology sub-study leads, co-Chief Investigators and the Trial Statisticians. Given time and funding limitations, no additional group could be analysed.

Similarly, a decision as to whether to assay all or a subset of follow-up nasopharyngeal samples using either the TF CARD multiplex or meta-serotyping will be taken on the basis of the baseline results. Given time and funding limitations, the decision was made not to assay further post-baseline samples.
